# Gel Electrolytes in the Development of Textile-Based Power Sources

**DOI:** 10.3390/gels11060392

**Published:** 2025-05-27

**Authors:** Ana Isabel Ribeiro, Cátia Alves, Marta Fernandes, José Abreu, Fábio Pedroso de Lima, Jorge Padrão, Andrea Zille

**Affiliations:** Centre for Textile Science and Technology (2C2T), Department of Textile Engineering, University of Minho, Campus of Azurém, 4800-058 Guimarães, Portugal; catia.alves@2c2t.uminho.pt (C.A.); marta.fernandes@det.uminho.pt (M.F.); id11556@uminho.pt (J.A.); fabioplima.conceicao@gmail.com (F.P.d.L.); padraoj@2c2t.uminho.pt (J.P.); azille@2c2t.uminho.pt (A.Z.)

**Keywords:** gel electrolytes, wearable electronics, e-textiles, energy storage, sensors

## Abstract

The interest in flexible and wearable electronics is increasing in both scientific research and in multiple industry sectors, such as medicine and healthcare, sports, and fashion. Thus, compatible power sources are needed to develop secondary batteries, fuel cells, supercapacitors, sensors, and dye-sensitized solar cells. Traditional liquid electrolytes pose challenges in the development of textile-based electronics due to their potential for leakage, flammability, and limited flexibility. On the other hand, gel electrolytes offer solutions to these issues, making them suitable choices for these applications. There are several advantages to using gel electrolytes in textile-based electronics, namely higher safety, leak resistance, mechanical flexibility, improved interface compatibility, higher energy density, customizable properties, scalability, and easy integration into manufacturing processes. However, it is also essential to consider some challenges associated with these gels, such as lower conductivity and long-term stability. This review highlights the application of gel electrolytes to textile materials in various forms (e.g., fibers, yarns, woven, knit, and non-woven), along with the strategies for their integration and their resulting properties. While challenges remain in optimizing key parameters, the integration of gel electrolytes into textiles holds immense potential to enhance conductivity, flexibility, and energy storage, paving the way for advanced electronic textiles.

## 1. Introduction

The rapid growth of wearable and portable smart technologies has significantly increased the demand for efficient and miniaturized energy storage systems [1,2,3,4,5,6]. In this field, fiber-based wearable electronics represent a promising intersection between textiles and technology, offering a wide range of opportunities for creating intelligent and interactive materials that can be integrated into daily life and technical products. These materials have attracted attention from scientists and industries in several areas due to their high flexibility, stretchability, and conformal contact with the skin [7,8]. Produced using well-established and cost-effective textile production methods, they offer the potential to create innovative and functional wearable devices with diverse applications in flexible sensors, personalized healthcare, entertainment, and thermal management [9]. Traditional energy storage devices, such as batteries, can hold significant energy but struggle to deliver it rapidly due to their high internal resistance. In contrast, capacitors can charge and discharge quickly but have limited energy storage capacity. To bridge this gap, the development of high-energy-density capacitors, known as supercapacitors, has emerged as a key research focus in electrochemical energy storage and conversion [10]. Supercapacitors stand out as one of the most promising energy storage solutions, relying on non-Faradaic and/or Faradaic processes occurring at the electrode–electrolyte interface. Faradaic processes involve electron transfer and redox reactions at the electrode surface, while non-Faradaic processes involve no electron transfer, only physical phenomena like double-layer charging or adsorption [11]. Compared to conventional batteries, supercapacitors offer notable advantages, including an extended lifespan of over 10,000 charge/discharge cycles, rapid charging within seconds, and superior power density. However, their practical use remains constrained by their relatively low energy density compared to batteries. Despite this limitation, ongoing research actively explores innovative strategies to enhance supercapacitor performance [1,12]. Supercapacitors can be classified into three types based on their energy storage mechanisms: electrical double-layer capacitors (EDLC), pseudocapacitors, and hybrid supercapacitors [12]. EDLCs function similarly to lithium-ion (Li-ion) batteries, storing energy through static charge accumulation caused by the adsorption of anions and cations onto the electrode surface. Typically, they are made from carbon-based materials like graphene or carbon nanotubes (CNTs), as their porous structures provide a high surface area, enabling greater electron activity and increased capacitance. In contrast, pseudocapacitors utilize Faradaic and electrochemical reactions between the electrolyte and the active electrode material for energy storage and are often composed of conductive polymers or metal oxides. Hybrid supercapacitors integrate EDLC and pseudocapacitor properties, making them particularly suitable for textile-based energy storage applications. Thus, selecting the appropriate materials for textile-based energy storage is crucial, as the final device must exhibit high conductivity, surface area, efficient electron transport, and strong power output while maintaining flexibility, stretchability, mechanical durability, and washability [13,14]. Electrolytes are essential components of electrochemical energy storage devices, where their physical and chemical characteristics significantly influence performance metrics such as capacity, power density, charge/discharge rate, cycle stability, and overall safety [15]. A widely accepted approach to enhancing the safety and flexibility of wearable energy storage systems is replacing liquid electrolytes with solid or quasi-solid-state electrolytes. Those electrolytes offer several advantages over other types, including high ionic conductivity, straightforward packaging, and the absence of liquid leakage. In the context of supercapacitors, polymer-based solid electrolytes are widely utilized and can be categorized into three main types: solid polymer electrolytes, gel polymer electrolytes, and polyelectrolytes. Gel polymer electrolytes, often referred to as quasi-solid-state electrolytes, contain a liquid phase [16,17]. These electrolytes consist of a polymer matrix, such as poly(ethyl oxide) (PEO), poly(ethylene glycol) (PEG), or poly(vinyl alcohol) (PVA), combined with an aqueous electrolyte like potassium hydroxide (KOH), sulfuric acid (H_2_SO_4_), or potassium sulfate (K_2_SO_4_), or a conducting salt dissolved in a solvent. Gel electrolytes commonly have the highest ionic conductivity compared to the three types of solid-state electrolytes due to a liquid phase [15,18]. However, inadequate wetting at the interface between the polymer gel electrolyte and the electrode may lead to poor electrochemical performance [19]. Selecting a host polymer compatible with the redox couples is key; this involves factors such as its pH sensitivity, low solubility, thermal stability, and mechanical properties [20]. Various techniques and material combinations can be employed to incorporate gel electrolytes into textiles, with the selection depending on the specific application requirements and the desired properties of the final product. This review examines recent advancements in textile-based energy storage systems that apply gel electrolytes, focusing on how their design and integration impact system performance. The discussion is structured according to different textile structures, including fibers, yarns, woven fabrics, knitted fabrics, and non-woven materials. Additionally, this review highlights the crucial role of gel electrolytes in preserving the durability of conductive properties, ensuring long-term electrochemical stability, mechanical flexibility, and overall device reliability.

## 2. Gel Electrolytes Applied in Textile Structures

### 2.1. Fibers

**Figure 1:**
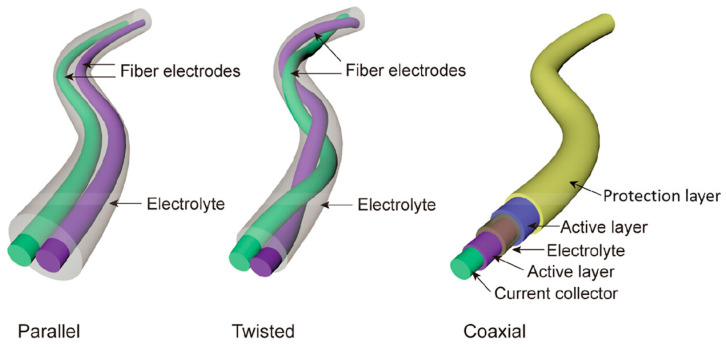
Scheme showing design of three common fiber supercapacitors using gel electrolytes [24].

In the field of wearable energy storage, fiber-form devices have received tremendous interest due to being highly flexible, lightweight, and easily integrated or directly weaved/knitted into textiles [21,22]. In this section, we will describe scientific studies that use fibers as a base for constructing energy storage textiles, focusing on their design, fabrication methods, and performance characteristics (Table 1). Flexible fiber-shaped supercapacitor devices are usually assembled in three different configurations, namely, parallel, twisting, and coaxial, and coated with an electrolyte (Figure 1) [23,24]. Various carbon-based materials have been studied for the development of these devices, including graphene and CNT, and their composites with transition metal oxides or conducting polymers, such as polyaniline (PANI) and poly(3,4-ethylenedioxythiophene) poly(styrenesulfonate) (PEDOT:PSS). These materials endow the fabricated supercapacitors with outstanding electrochemical attributes, including high specific capacitance and electrical conductivity, porosity, and reversible electrochemical doping/de-doping capability [25].

**Figure 2:**
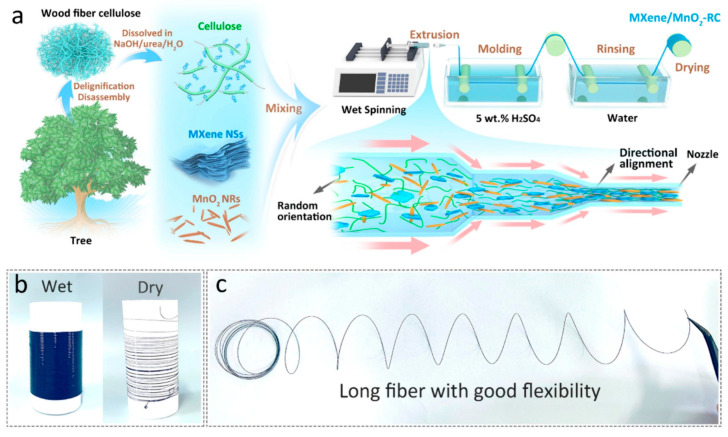
(**a**) Scheme depicting fabrication process of MXene/MnO_2_-RC composite fibers. (**b**) Image showing fibers in both wet and dry conditions. (**c**) Long fiber demonstrating excellent flexibility [45].

Among the various electrode materials, graphene has emerged as a promising candidate for the creation of flexible fibrous supercapacitors [26,27]. Gao et al. [28] reported the creation of a wearable fiber-shaped supercapacitor using reduced graphene oxide (rGO) obtained via a wet-spinning method. The graphene oxide (GO) dispersions were injected into a coagulation bath containing calcium chloride (CaCl_2_) and dried. This was followed by chemical reduction by immersion into a hydriodic acid solution. Then, to prepare the fiber-shaped supercapacitor, two symmetric rGO fibers were coated with PVA/H_2_SO_4_ gel electrolyte and twisted to form a double-helix-shaped fiber. The produced rGO fibers showed a highly oriented porous structure, facilitating ion transfer and improving ionic site accessibility. The device displayed a volume ratio capacitance of 5.0 mF/cm^3^ and observed an increase in the specific capacitance tested at 40 mA/cm^3^, which could be attributed to the wetting of graphene with the electrolyte during the charge/discharge cycle. Moreover, the assembled supercapacitors successfully filtered an alternating current (AC) input signal of 1.5 V (60 Hz) to a stable direct current (DC) output signal of 1.04 V. In another work from Park et al. [29], hybrid fibers of porous CNT-rGO were also fabricated by wet spinning. CNTs were used as spacers between the rGO sheets, enhancing the porosity of rGO and the ion accessibility. The hybrid fibers were subsequently activated hydrothermally using hydrogen peroxide (H_2_O_2_) and ammonium hydroxide (NH_4_OH), further enhancing the porosity. Two parallel individual fibers were coated with a thin layer of the PVA/KOH gel electrolyte to form the solid-state fiber-based supercapacitors, where the volumetric capacitance reached 6.1 × 10^4^ mF/cm^3^ and the volumetric energy density reached 4.83 mWh/cm^3^. The supercapacitors also exhibited excellent cycling stability of 94% after 10,000 cycles and stable capacitive performance under deformation conditions. Zhang et al. [30] demonstrated the application of zinc-ion (Zn-ion) fiber supercapacitors in wearable electronics by hydrothermally assembling rGO and single-walled CNT fiber as a cathode, coating graphite fibers with a uniform Zn layer as an anode, and producing a new neutral zinc sulfate (ZnSO_4_)-filled polyacrylic acid (PAA) hydrogel as a quasi-solid-state electrolyte. The single-walled CNTs were added to prevent the restacking of rGO nanosheets, so the ion-accessible surface was enlarged, while the hydrogel electrolyte offered high ionic conductivity and stretchability. The zinc (Zn) fiber supercapacitors delivered a high energy density of 4.9 × 10^−2^ Wh/cm^3^ at a power density of 1.8 × 10^−1^ W/cm^3^, a volumetric capacitance of 1.0 × 10^5^ mF/cm^3^ at a current density of 400 mA/cm^3^, and an outstanding stable cycling stability of 98.5% capacity retention after 10,000 cycles, showing promising applications in wearable electronics. In the work carried out by Li et al. [31], the porosity of rGO was increased by doping with 1T-molybdenum disulfide (MoS_2_). MoS_2_ prevents the tight restacking of rGO into compact lamellar structures. The fibers were prepared by wet spinning, followed by reduction via immersion in aqueous hydroiodic acid (HI) solution. They were then treated with PVA/phosphoric acid (H_3_PO_4_) gel electrolytes to produce all-solid-state fiber-shaped supercapacitors. The volumetric capacitance reached 2.2 × 10^5^ mF/cm^3^ (at 50 mA). Three supercapacitors were connected in parallel in a fabric, reaching a high capacitance of 2.4 × 10^2^ mF/cm^2^ (at 500 mA) and an energy density of 32 mWh/cm^3^. However, the specific capacity was reduced by 28% after bending 500 times at different angles. Chen and coworkers [32] fabricated a new type of 1D carbon nanomaterial, carbon nanobranches, covered with abundant carbon dots by the pyrolysis of starch. The conductive fibers composed of carbon nanobranches and thermoplastic polyurethane (PU) were produced using a customized microfluidic spinning approach. For the assembly of a microsupercapacitor, two aligned conductive fibers were twisted together and coated with the PVA/H_3_PO_4_ gel electrolyte. The produced fiber-shaped supercapacitor displayed a large specific capacitance of 2.0 × 10^2^ mF/cm^2^, a high energy density of 4.5 × 10^−6^ Wh/cm^2^ (at 8.0 × 10^−5^ W/cm^2^), and impressive electrochemical stability with a capacitance retention of 98% after 10,000 cycles. Furthermore, after integration into a woven fabric, it successfully powered 19 light-emitting diode (LEDs). The development of supercapacitors based on composites of carbon materials and PANI has also been extensively growing. PANI has appreciable electrochemical properties such as high pseudocapacitance (around 1200 F/g), cost-effectiveness, and environmental stability [33,34]. Storage efficiency depends on the degree of H^+^ doping and the oxidation state of the macromolecular chain, and so grafting PANI onto carbonaceous materials enables ion transfer during the redox reaction, which increases the capacitance [35]. For example, Jiang et al [36] designed supercapacitor fibers based on PANI, GO, and CNTs for wearable electrodes. First, a composite film was developed by preparing a CNT film by chemical vapor deposition, followed by immersion in a GO/PANI mixture solution. The composite film exhibited a specific capacitance of 729.3 F/g (5.1 × 10^2^ mF/cm^2^) at 1 A/g. Then, for the preparation of the fiber-like supercapacitor, the composite film was over-twisted into two helical fibers and coated with a PVA/H_3_PO_4_ gel electrolyte as the separator. The two helical fibers were intertwined and coated with the PVA/H_3_PO_4_ gel electrolyte again, creating stretchable fiber-shaped supercapacitors. This device presented a specific capacitance of 89.5 F/g (1.8 × 10^2^ mF/cm^2^) and a relatively good capacitance retention of approximately 80% over 5000 cycles (at 5 A/g). In another study, Adusei et al. [21] developed PANI–graphene–CNT hybrid fiber electrodes by preparing CNT/rGO via the chemical vapor deposition of rGO in CNT fibers, followed by coating with PANI nanorods via oxidation polymerization. The hybrid fibers were used as the supercapacitor electrode using the gel electrolyte polyvinylidene fluoride (PVDF)- 1-ethyl-3-methylimidazolium tetrafluoroborate (EMIMBF_4_). The fast-redox reactions between the PANI and the electrolyte gave rise to the superior electrochemical performance of these fibers. The supercapacitors achieved a gravimetric energy density of 1.3 × 10 Wh/kg and a power density of 1.4 × 10^3^ W/kg (at a current density of 1 A/g). In addition, the supercapacitors demonstrated excellent cyclic stability by maintaining a high capacitance retention of 98.4% after 2000 cycles. PEDOT is another conducting polymer that has drawn interest in the development of flexible supercapacitors owing to its high conductivity (up to 103 S/cm), substantial theoretical specific capacitance (210 F/g), and high chemical and physical stability [37]. For example, He et al. [38] synthesized PEDOT:PSS thin-walled hollow fibers by coaxial extrusion via wet spinning to obtain a flexible supercapacitor with a high surface area. The thin fibers displayed a diameter of approximately 125 μm and a wall structure of 8 μm, which allowed for an increase in the specific capacitance of ~ 67%. When used as electrode materials for gel electrolyte fiber-shaped supercapacitors, the fibers displayed a capacitance of 1.1 × 10^2^ mF/cm^2^ (at 0.3 mA/cm^2^) with a high energy density of 9.0 × 10^−6^ Wh/cm^2^ (at 1.1 × 10^−4^ W/cm^2^). In addition, impressive long-term cycle stability was observed, maintaining 81% of the initial capacitance after 10,000 cycles. The excellent electrochemical performance was attributed to the fiber’s structure, the electrochemical characteristics of the PEDOT:PSS, and the stability of the gel electrolyte. In another work from Gibertini et al. [39], an easy method was developed for the preparation of robust and flexible composite fiber-shaped capacitors by wet spinning nanofibrillated Kevlar^®^, followed by immersion in a gel state with PEDOT:PSS aqueous dispersion. The symmetric device, coupled with PVA/H_3_PO_4_ electrolyte gel, displayed a capacitance of 1.1 mF, a volumetric energy density of 7.1 × 10^−2^ mWh/cm^3^, and good capacitance retention of 80.5% after 10,000 cycles. When integrated into an e-textile circuit, it was able to power a blue LED for several seconds. Efforts have been made to produce long and continuous fibers electrodes. Hong et al. [40] designed a multichannel spinning method to continuously produce supercapacitor fibers, kilometers in length, at high production rates of up to 118 m/h. A multichannel spinneret, with two internal parallel nozzles for electrode inks (PEDOT:PSS/CNT) and a larger external nozzle carrying gel electrolyte (PVA/chitosan), simultaneously extruded two fiber electrodes into the coagulation bath (sodium hydroxide (NaOH) containing lithium perchlorate (LiClO_4_)), where the gel electrolyte was quickly solidified. The supercapacitor fibers displayed good electrochemical stability after being bent for 1 × 10^5^ cycles. In particular, they had a volumetric capacitance of 1.6 × 10^4^ mF/cm^3^ (at 0.11 A/cm^3^) and displayed cycle stability, with 96% capacity remaining after 5000 cycles. Moreover, it could be woven into a flexible and wearable power scarf, reaching a capacitance of 1.34 × 10^6^ F and an energy of 116 Wh. In the work by Lai and coworkers [37], a new strategy was developed to synthesize wire-shaped solid-state supercapacitors with excellent electrochemical and mechanical performances in a facile dip-coating process. Polyacrylonitrile (PAN) nanofibers were electrospun on a titanium (Ti) metal wire using glycerol to form a sacrificing aerogel template with a huge void volume, followed by dip-coating with PEDOT:PSS and the dissolution of the template. PSS was then removed using H_2_SO_4_, forming a highly porous PEDOT layer. Supercapacitors based on the Ti/PEDOT wire electrodes and PVA/H_2_SO_4_ gel electrolyte reached an energy density of 5.5 Wh/kg (at 3.5 × 10^2^ W/kg), exhibiting a specific capacitance of 68 F/g (at 0.97 A/g) and a power density of 9.1 × 10^3^ W/kg (at 4.6 Wh/kg). The cycling stability of the fabricated supercapacitor retained 81% of the capacitance after 10,000 cycles (at 25 A/g). Moreover, the use of supercapacitors as a power source in wearables was demonstrated, as they could successfully power a purple LED by knitting it into a fabric and connecting it in series. Transition metal oxides such as vanadium pentoxide (V_2_O_5_) were incorporated into conductive polymeric fibers to achieve high capacitance and good cyclic stability, protect the structure from damage during charge/discharge cycles, and increase the molecular order and stability. Achieving high performance using wet spinning, Xu et al. [41] adopted a direct mixing process to fabricate supercapacitors successfully based on PEDOT:PSS/V_2_O_5_ hybrid fibers. The fiber-shaped supercapacitor in the PVA/H_2_SO_4_ gel electrolyte achieved an energy density of 1.4 × 10^−6^ Wh/cm^2^ (at 2.0 × 10^−5^ W/cm^2^), a specific capacitance of 6.0 × 10 mF/cm^2^, and excellent cycle stability with a retention of 94.02% after 4000 cycles (at 0.1 mA/cm^2^). MXenes are 2D transition metal carbonitride layered materials that have attracted considerable interest for the development of energy storage devices because of their advantageous features, such as large specific surface area, hydrophilicity, high conductivity (>6000 S/cm), adjustable functional terminal groups, and fast electron transport capabilities [42]. Qin et al. [43] used Mo_1.33_C *i*-MXene nanosheets and PEDOT:PSS as the base materials to prepare composite high-performance fibers as electrodes that can be applied to next-generation wearable devices. PEDOT:PSS was used as a conductive binder to promote spinnability and improve flexibility by increasing the interlayer spacing between Mo_1.33_C *i*-MXene layers. This new fiber was assembled into a solid-state fiber-based asymmetric supercapacitor with reduced rGO fibers and a thin layer of PVA/H_2_SO_4_ gel electrolyte, reaching a capacitance of 105 F/g and an energy density of 3.7 × 10^−2^ Wh/g (at 1.6 V). The supercapacitor also demonstrated excellent cycling stability, with a capacitive retention of 94% after 10,000 cycles. In another work, Lu et al. [44] developed ternary manganese dioxide (MnO_2_)/MXene-Ti_3_C_2_T_x_/rGO composite fiber supercapacitors by wet spinning MXene-Ti_3_C_2_T_x_/GO, reducing it in HI/acetic acid (CH_3_COOH) solution, and immersing it in potassium permanganate (KMnO_4_) solution. Two of these fiber electrodes were twisted together and assembled with a PVA/LiCl gel electrolyte in an all-solid-state symmetric fiber supercapacitor. The new fiber supercapacitor displayed a volumetric capacitance of 2.4 × 10^4^ mF/cm^3^, a volumetric energy density of 2.1 × 10^−3^ mWh/cm^3^ (at 8.2 × 10^−23^ W/cm^3^), and a good cycle stability of 92% after 10,000 cycles. Wei et al. [45] prepared regenerated cellulose (RC)-based conductive microfibers in a simple, continuous, and scalable wet-spinning process. A mixture of MXene/MnO_2_-wood fiber cellulose (WFC) was span in an H_2_SO_4_ coagulation bath; we obtained a MXene/MnO_2_-RC fiber cathode with a highly aligned and stable three-dimensional (3D) nanonetwork structure formed by the self-assembly of RC during molecular chain rearrangement. Due to the strong interaction between cellulose and electroactive nanomaterials and their highly aligned structure, the conductive fibers showed good tensile strength and improved electrolyte permeation. The assembled quasi-solid-state fiber electrodes, with MXene/MnO_2_-RC as the cathode, Zn wire as the anode, and zinc sulfate (ZnSO_4_)/gelatin gel as the electrolyte, showed excellent electrochemical performance with a high volumetric capacitance of 1.1 × 10^2^ mF/cm^3^ (at 0.57 mA/cm^3^), an energy density of 2.2 × 10^−2^ mWh/cm^3^ (at 0.57 A/cm^3^), excellent cycling stability with 90.5% capacity retention, and close to 100% Coulombic efficiency, after 5000 cycles. Recently, Ovhal et al. [46] produced one-meter-long 3D fiber-shaped supercapacitors of MXene-PEDOT:PSS active electrodes using the 3D direct-ink-writing method. MXene/PEDOT:PSS/Ag/MXene/PEDOT:PSS composite fibers with a sandwich structure were fabricated by extruding the MXene/PEDOT:PSS ink in an ion-permeable dialysis membrane separator; then, an Ag current collector was printed on top of the electrode, followed by an additional layer of MXene/PEDOT:PSS. The process was repeated for five cycles. The PVA/H_3_PO_4_ gel electrolyte was then printed; we finished the process by encapsulating the whole fiber with NOA 63 resin (Figure 2). The Ag current collector was used to facilitate faster charge transport, eliminating the thickness and length problems of electrode-dependent capacitance in fiber-shaped devices. The fiber-shaped 3D supercapacitors showed prominent electrical conductivity of 1.6 × 10^3^ S/cm, a high areal capacitance of 1.1 × 10^3^ mF/cm^2^, a gravimetric capacitance of 185.9 F/g, and a high areal energy density of 9.4 × 10^−5^ Wh/cm^2^ (at 1.1 × 10^−3^ W/cm^2^). Moreover, the fibers demonstrated an excellent stability of 92% after 25,000 cycles. After being incorporated into a woven fabric, they were able to successfully power an LED indicator.

Smart textiles can behave like batteries by incorporating flexible fiber-shaped rechargeable materials on a single-fiber architecture with high energy density and long-term stability. To ensure that a thin fiber battery works upon stretching, bending, and cleaning, Xiao et al. designed a high-performance rechargeable solid-state Zn/MnO_2_ fiber battery with a GO-embedded PVA hydrogel electrolyte. GO supports ion transport, improving the ionic conductivity of the hydrogel electrolyte. This rechargeable solid-state fiber battery showed stable cyclic performance, exceeding the standard performance with up to 500 h of 98.0% capacity after more than 1000 charging/recharging cycles. This Zn/MnO_2_ fiber battery could be used as a stable energy power unit for human health monitoring by seamlessly integrating it into a multifunctional e-textile [47]. Zhang et al. [48] developed high-voltage coaxial–fiber aqueous rechargeable Zn-ion batteries (CARZIBs) with outstanding flexibility and high-capacity retention. This novel coaxial structure consisted of Zn nanosheet array on CNT fibers as the core electrode, carboxymethyl cellulose (CMC)-ZnSO_4_ as the gel electrolyte, and Zn hexacyanoferrate (ZnHCF) on a CNT sheet as the outer electrode. CMC, used as a cathode binder, provided good mechanical properties. The assembled battery achieved a large capacity of 100.2 mAh/cm^3^ (at 0.1 A/cm^3^) and a high energy density of 195.4 mWh/cm^3^ (at 2.0 × 10^−1^ W/cm^3^), a capacity retention of 91.8% after 200 cycles, and a Coulombic efficiency of 96.8%. Additionally, the wearability indicated by the high-capacity retention of 93.2% (after bending 3000 times) was demonstrated by incorporating serial and parallel CARZIB devices into a fabric to illuminate a 3.3 V blue LED. Pan et al. [49] successfully designed advanced cathodes of CNT-stitched pyrovanadate (ZVO) 2D nanosheets, grown on oxidized CNT fibers, for wearable quasi-solid-state ZIBs. CNTs improved electronic conductivity and mechanical robustness across the 2D cathode nanosheets. Moreover, the large open frameworks of ZVO offer superior reversible Zn^2+^ ion deintercalation/intercalation. The hybrid cathode was assembled with Zn nanosheets, electrodeposited onto CNT fibers as the anode and CMC/HSO_4_ as the electrolyte gel. The fiber-shaped high-performance ZIB includes a high-rate capability of 69.7% capacity retention at 100 times enhancement in current density (discharge in 25 s), a high stack volumetric energy density of 7.2 × 10^−2^ mWh/cm^3^, and excellent flexibility with cycle stability of 88.9% retention after 2000 cycles (at 1.0 A/cm^3^). The fiber-shaped ZIB knitted into a cotton (CO) string glove exhibited outstanding flexibility after bending to different states. Li et al. [50] made a great advance in the further development of Ag-based cathodes for flexible batteries by eliminating issues associated with the structural pulverization and the migration of Ag^+^ ion into the electrolyte solution. The cathode of the quasi-solid-state fiber-shaped zinc silver oxide (Zn-Ag_2_O) battery was constructed by depositing Ag_2_O nanoparticles onto a nitride-doped CNT hybrid fiber and coating a conducting polymer with a protective layer of PEDOT:PSS as the cathode to eliminate the issues mentioned. The anode was obtained by growing Zn nanosheets directly on the hybrid fiber and PVA/KOH flexible hydrogel was employed as an electrolyte. The as-prepared flexible fiber-shaped Zn–Ag_2_O battery could be integrated with a wind power generator to be charged solely by wind energy, exhibiting a high capacity of 1.05 mAh/cm^2^, a high energy density of 1.6 × 10^−3^  mWh/cm^2^, a remarkable power density of 1.4 × 10^−3^ W/cm^2^, and good electrochemical durability with 79.5% retention after 200 cycles. In another work from Li et al. [51], ZIBs with a double-helix structure were reported and used for highly deformable wearable textiles. Ag fiber was covered in graphene nanosheets to protect it from corrosion and coated with PANI to enhance the electrical conductivity (cathode). Another Ag fiber was coated with Zn nanoflake by electrodeposition (anode). Then, both electrodes and the PVA/ZnSO_4_ gel electrolyte were encapsulated with a PU film, which was parallel-twisted around a spandex fiber. The resulting fiber-shaped Zn-ion battery displayed an enhanced specific capacity of 32.56 mAh/cm^3^, a desirable energy storage performance with an energy density of 3.6 × 10^−2^ mWh/cm^3^ (at 3.0 × 10^−3^ W/cm^3^), excellent cycling, and deformation stability with a capacity of 76.5% after 1000 cycles, and capacities of 99, 93.6, and 91.5% during knotting, bending, and twisting, respectively. Due to the high stretchability of the spandex fiber and the unique helix structure, the battery exhibited a remarkable strain up to 900%, maintaining 71% of its original capacity. Another stretchable fiber-shaped battery was reported by Xiong et al. [52], where aqueous Al-ion batteries were assembled with a manganese hexacyanoferrate (MnHCF) cathode, a GO-modified MoO_3_ anode, and a PVA/aluminiumtrifluormethansulfonat (Al(CF_3_SO_3_)_3_) hydrogel electrolyte. A CNT fiber was coated with the active materials within the hydrogel electrolyte. Then, both fibers were assembled in parallel, winded on an elastic silicone rubber substrate to form a helical structure, and coated with another layer of the gel electrolyte. PVA was employed due to the good mechanical properties and high-water absorbency, which resulted in a PVA-based hydrogel with good transparency, high modulus (0.55 MPa) and strain (461%), and ionic conductivity (2.2 × 10^−2^ S/cm), indicating the potential of the hydrogel for improving the electrochemical performance of aluminum (Al) ion batteries. The assembled fiber-based battery achieved a high specific capacity of 42 mAh/cm^3^, a high energy density of 3.1 × 10^−2^ Wh/cm^3^ (at 0.5 A/cm^3^), and good stability by retaining 91.6% capacity after 100 cycles (at 1 A/cm^3^). Finally, when integrated into wearable textiles, the developed fiber-shaped batteries could power one red LED, showing their potential for wearable electronics. Recently, Zhao et al. [53] developed a new method for preparing flexible fiber Li-ion batteries using direct ink writing-based 3D printing. Lithium iron phosphate (LFP) and lithium titanate (LTO) were used as cathode and anode active materials, respectively. First, GO was etched using H_2_O_2_ and reduced with vitamin C, followed by freeze-drying to obtain rGO with holes (HrGO). This porous structure provides additional channels, increasing the Li^+^ ions’ transmission rate. An adhesive based on PVDF was prepared by dissolving PVA in *N-*Methyl-2-pyrrolidone (NMP), and then PVDF was added. For the preparation of the printing ink, LFP or LTO and CNTs were dissolved in the PVA/PVDF solution with a specific mass ratio of LFP/LTO: HrGO:CNTs:PVA/PVDF of 12:3:1:5. The flexible fiber electrodes were produced by extruding the printing inks into a sodium metaborate (NaBO_2_) aqueous solution. BO_2_^-^ ions undergo solvent exchange with NMP and transfer to the interior of the electrode, precipitating the PVDF on the surface of the fibers and cross-linking the PVA, which forms a three-dimensional network structure with borate ester bonds, improving mechanical properties and stability. Moreover, the dynamic reversibility of the borate bond imparts a self-healing property to the material. The fibers then passed through a PVDF/HFP/aluminum oxide (Al_2_O_3_) solution, forming a separator layer on the surface of the fiber electrode, which prevents the passage of electrons while allowing ions to pass through. The obtained electrodes reached a strain of ~30%, and the assembled fiber Li-ion battery exhibited a volumetric energy density of 1.6 × 10^−1^ Wh/cm^3^. The functional fibers and developed materials were applied in wearable AC line filters [28], wearable and portable miniaturized energy storage devices [29,30,36,44,48,49,50,51,52], and textile capacitors [21,32,39]. The examples presented here, and summarized in Table 1, show the trend towards the use of gel electrolytes in the development of fiber-based electrodes. This fact is explained by PVA’s ability to form stable films and its low economic cost [54]. However, due to its low electrical conductivity at ambient temperature, it is usually used in complexes with acids such as H_2_SO_4_ and H_3_PO_4_, which provide free ions in gel electrolytes, acting as charge carriers [55]. In the case of PVDF, when compared to other polymers, it presents higher mechanical properties and excellent chemical and thermal stability, being suitable for use as the skeleton of gel electrolytes. PVDF, when mixed with HFP or ionic liquids, has a reduced crystalline phase and increased amorphous region, increasing ion permeation capacity [56].

Analyzing the overall results (Table 1), gel electrolytes based on PVA, PVDF, CMC, or gelatin have been widely utilized as polymeric matrices for the development of fiber-based energy storage materials, with PVA emerging as the most frequently used. PVA is highly favored due to its excellent mechanical flexibility, film-forming ability, high ionic conductivity, water solubility, and environmental friendliness, making it ideal for wearable and flexible energy devices. The PVA matrix can be effectively combined with various acids and salts such as H_2_SO_4_, H_3_PO_4_, LiCl, ZnSO_4_, and KOH, as well as natural polymers like chitosan, to enhance ionic conductivity and electrochemical stability. Additionally, the incorporation of GO further improves performance by increasing ion transport and mechanical strength. These gel electrolytes are mostly applied in conductive fibers such as CNT, GO, and PEDOT:PSS, or in other synthetic fibers like PAN, Kevlar^®^, and spandex.

**Table 1:** Comparison of fiber-based energy storage devices using gel electrolytes, highlighting preparation methods and electrochemical properties.

Gel Electrolyte	Fiber Composition	Electrodes	Preparation Method	Conductivity Tests and Results	Ref.
PVDF/EMIMBF_4_	CNT	PANI–graphene–CNT	Dry-spinning CNT fibers; CNT/rGO via chemical vapor deposition; PANI nanorod coating; gel electrolyte coating	Energy density of 1.3 × 10 Wh/kg; power density of 1.4 × 10^3^ W/kg; 98.4% capacitance retention after 2000 cycles	[21]
H_2_SO_4_	GO	rGO	GO wet spinning; gel electrolyte coating and two-fiber twisting	Excellent performance and fast reponse rate (500 V/s); cycling stability; stable performance at different angles	[28]
KOH	CNT-GO	CNT-rGO	CNT-GO wet spinning; hydrothermal activation; gel electrolyte coating	Volumetric capacitance of 6.1 × 10^4^ mF/cm^3^; volumetric energy density of 4.8 × 10^−3^ Wh/cm^3^; 94% stability after 10,000 cycles; stable performance at different angles	[29]
ZnSO_4_-filled PAA	CNT-GO	CNT/rGO and Zn/graphite	Hydrothermally assemble CNT/rGO; coating of Zn fiber with graphite by electrodeposition; gel electrolyte coating	Ionic conductivity of 21.7 mS/cm; stretchability up to 2500; energy density of 48.5 mWh/cm^3^; volumetric capacitance of 1.0 × 10^5^ mF/cm^3^; 98.5% stability after 10,000 cycles	[30]
H_3_PO_4_	GO	rGO-MoS_2_	Wet spinning of MoS_2_-GO, followed by chemical reduction; gel electrolyte coating	Volumetric capacitance of 2.2 × 10^5^ mF/cm^3^; three fibers connected—capacitance of 2.4 × 10^2^ mF/cm^2^; energy density of 3.2 × 10^−2^ Wh/cm^3^; 72% stability after bending 500 times at different angles	[31]
H_3_PO_4_	Carbon nanobranches	Carbon nanobranches/PU	Carbon nanobranches covered with carbon dots by pyrolysis of starch; microfluidic spinning for carbon nanobranches/PU fibers; twisting of fibers and gel electrolyte coating	Specific capacitance of 2.0 × 10^2^ mF/cm^2^; energy density of 4.5 μWh/cm^2^; 98% stability after 10,000 cycles; powered 19 LEDs	[32]
H_3_PO_4_	CNT/GO/PANI	CNT/GO/PANI	CNT film by chemical vapor deposition; immersion in a GO/PANI mixture solution; over-twisting of strips of the film into two helical fibers; gel electrolyte coating; intertwining fibers and gel electrolyte coating	CNT/GO/PANI film with a specific capacitance of 5.1 × 10^2^ mF/cm^2^; specific capacitance of 1.8 × 10^2^ mF/cm^2^; 80% stability after 5000 cycles; stability after bending for 500 cycle at 180°	[36]
H_2_SO_4_	PAN	Ti/PEDOT	Electrospun PAN nanofibers on a Ti wire with glycerol; sip-coating with PEDOT:PSS; PSS etching with H_2_SO_4_; gel electrolyte coating, twisting, and coating again with gel electrolyte	Energy density of 5.5 Wh/kg; specific capacitance of 68 F/g; power density of 9.1 × 10^3^ W/kg; 81% stability after 10,000 cycles; stability under different deformations; powered one LED	[37]
LiCl	PEDOT:PSS	PEDOT:PSS	Coaxial PEDOT:PSS wet spinning; gel electrolyte coating	Electrical conductivity of 1514 S/cm; specific areal capacitance of 1.2 × 10^2^ mF/cm^2^; energy density of 9 × 10^−6^ Wh/cm^2^; 81% stability after 10,000 cycles; Coulombic efficiency of ~100%; stable specific capacitance after bending 3000 times at 180°	[38]
H_3_PO_4_	Kevlar^®^ fibers	PEDOT:PSS/Kevlar^®^	Wet spinning of nanofibrillated Kevlar^®^; immersion in PEDOT:PSS gel	Capacitance of 1.1 mF; volumetric energy density of 7.1 × 10^−2^ Wh/cm^3^; 80.5% stability after 10,000 cycles; stability under different deformations; Coulombic efficiency of 99.1%; powered one LED	[39]
Chitosan	PEDOT:PSS/CNT	PEDOT:PSS/CNT	Wet spinning of PEDOT:PSS/CNT with internal nozzles for electrode inks and external nozzle carrying the gel electrolyte	Volumetric capacitance of 1.6 × 10^4^ mF/cm^3^; 96% stability after 5000 cycles; electrochemical stability after 1 × 10^5^ bending cycles	[40]
H_2_SO_4_	PEDOT:PSS	PEDOT:PSS/V_2_O_5_	PEDOT:PSS/V_2_O_5_ wet spinning; gel electrolyte coating	Energy density 1.4 × 10^−6^ Wh/cm^2^; specific capacitance of 6.0 × 10 mF/cm^2^; 94.02% stability after 4000 cycles	[41]
H_2_SO_4_	PEDOT:PSS	PEDOT:PSS Mo_1.33_Ci-MXene and rGO fibers	PEDOT:PSS Mo_1.33_Ci-MXene and GO wet spinning; gel electrolyte coating	Capacitance of 105 F/g; energy density of 3.7 × 10^−2^ Wh/g; 94% stability after 10,000 cycles	[43]
PVA–LiCl	GO	MnO_2_/Ti_3_C_2_T_x_/rGO	Ti_3_C_2_T_x_/GO wet spinning; immersion in HI/CH_3_COOH; immersion in KMnO_4_ solution; twisting of fibers and gel electrolyte coating	Capacitance of 2.4 × 10^4^ mF/cm^3^; energy density of 2.1 × 10^−3^ Wh/cm^3^; 92% stability after 10,000 cycles; 100% stability after bending 1000 times at 90°	[44]
ZnSO_4_/gelatin		MXene/MnO_2_-RC (cathode) and Zn wire (anode)	MXene/MnO_2_-RC fiber cathode wet spinning; gel eletrolyte coating	Volumetric capacitance of 1.1 × 10^2^ mF/cm^3^; energy density of 2.2 × 10^−2^ Wh/cm^3^; 90.5% stability and 100% Coulombic efficiency after 5000 cycles	[45]
PVA/H_3_PO_4_		MXene/PEDOT:PSS/Ag/MXene/PEDOT:PSS	MXene/PEDOT:PSS ink by 3D direct-ink writing; printing of Ag on top of the electrode; printing the gel electrolyte; Coating in NOA 63 resin	Electrical conductivity of 1.6 × 10^3^ S/cm; areal capacitance of 1.1 × 10^3^ mF/cm^2^; gravimetric capacitance of 185.9 F/g; areal energy density of 9.4 × 10^−5^ Wh/cm^2^; 92% stability after 25,000 cycles; powered one LED	[46]
PVA/GO/ZnSO_4_/MnSO_4_	Carbon	Carbon wire coated with nano-MnO_2_ (cathode) and Zn wire (anode)	GO gel crosslinking with nano-MnO_2_; gel eletrolyte coating; silicone layer coating	Gel electrolyte with ion conductivity of 2.1 × 10^−2^ S/cm; ~230% stretchability and self-healing; energy density of 91 Wh/L; 98.0% stability after 1000 cycles	[47]
CMC/ZnSO_4_	CNT fiber	CNT fiber coated with ZnHCF (cathode); CNT fiber coated with Zn nanosheet arrays (anode)	Roll electrodeposition of Zn arrays on CNT fibers; gel electrolyte coating comprising CNT sheets, ZnHCF, and CNT sheets	Capacity of 100.2 mAh/cm^3^; energy density of 2.0 × 10^−1^ Wh/cm^3^; 91.8% stability after 200 cycles; 96.8% Coulombic efficiency; 93.2% stability after bending 3000 cycles at 90°; powered one LED	[48]
CMC-SO_4_	CNT fiber	CNT fiber coated with stitched ZVO nanosheets (cathode) and CNT fiber coated with Zn (anode)	CNT-stitched ZVO nanosheets hydrothermally; electrodeposition of Zn on CNT fiber; gel electrolyte coating; twisting of electrodes	69.7% stability after 100 cycles; volumetric energy density of 7.2 × 10^−2^ Wh/cm^3^; 88.9% stability retention after 2000 cycles; 100% stability after bending angles from 0 to 180°	[49]
KOH	CNT fiber	Nitride-doped CNT fiber coated with Ag_2_O/PEDOT:PSS (cathode) and CNT fiber coated with Zn (anode)	Deposition of Ag_2_O on a nitride-doped CNT fiber and coating with PEDOT:PSS; electrodeposition of Zn on CNT fiber; gel electrolyte coating; twisting of two electrodes	Capacity of 1.05 mAh/cm^2^; energy density 1.6 × 10^−3^ Wh/cm^2^; power density of 1.4 × 10^−3^ W/cm^2^; 79.5% eletrochemical stabily retention after 200 cycles	[50]
ZnSO_4_	Ag fiber Spandex fiber	Ag fiber coated with graphene and PANI (cathode) and Ag fiber coated with Zn (anode)	Coating of Ag fibers with graphene and PANI, and Zn nanoflakes by electrodeposition; gel electrolyte coating; encapsulation with PU, and twisting around a spandex fiber	Specific capacity of 32.56 mAh/cm^3^; energy density of 3.6 × 10^−2^ Wh/cm^3^; stability of 76.5% after 1000 cycles and 99.0, 93.6 and 91.5% during knotting, bending, and twisting; strain up to 900%	[51]
Al(CF_3_SO_3_)_3_	CNT fiber	CNT fiber coated with MnHCF (cathode) and CNT fiber coated with GO/MoO_3_ (anode)	Coating of CNT fibers with MnHCF and GO/MoO_3_; gel electrolyte coating; assembled on a silicone rubber substrate and gel electrolyte coating	Al(CF_3_SO_3_)_3_ hydrogel with ionic conductivity of 2.2 × 10^−2^ S/cm and strain of 461%; specific capacity of 42 mAh/cm^3^; energy density of 3.1 × 10^−2^ Wh/cm^3^; stability of 91.6% after 100 cycles	[52]
PVDF/HFP/Al_2_O_3_		LFP (cathode) and LTO (anode)	Etching of GO with H_2_O_2_ and reduction with vitamin C; PVA/PVDF adhesive in NMP; printing ink by dissolving LFP or LTO and CNTs in the PVA/PVDF solution; extrusion into a NaBO_2_ solution; immersion in electrolyte	Specific capacity/capacity retention rate of 153.7 mAh/g/92% and 156.5 mAh/g/86.32% after 100 cycles, LFP/HrGO and LTO/HrGO fiber electrodes; FLIB@HrGO with a discharge capacity/capacity retention rate of 142.2 mAh/g/90.4%, after 100 cycles; specific capacity of 62.42 mAh/cm^3^; energy density of 1.6 × 10^−1^ Wh/cm^3^	[53]

### 2.2. Yarns

The preparation of yarn-based power sources using gel electrolytes has become a prominent research topic in recent years in the development of batteries and supercapacitors (Table 2). Similar to what can be observed for fibers, the use of yarns to assemble energy storage textiles is also linked to their versatility, applicability, and their ease of integration into the device. Here, we describe the latest scientific studies where different yarns were used in combination with gel electrolytes to develop energy storage apparatuses, highlighting the assembly procedure, components, and electrochemical performance. Yarns from different textile substrates can be used, from polyester (PET) to cotton (CO) or carbon fibers, which can then be assembled into yarn supercapacitors (YSCs). The most widely used method to assemble energy storage devices using yarns involves the construction of two electrodes, an anode and a cathode, by dip-coating, electrodeposition, electroless deposition, or in situ polymerization. The yarns are then used to assemble the devices, combining them with different gel electrolytes, resulting in high-energy and high-power-density devices with superior electrochemical performances.

**Figure 3:**
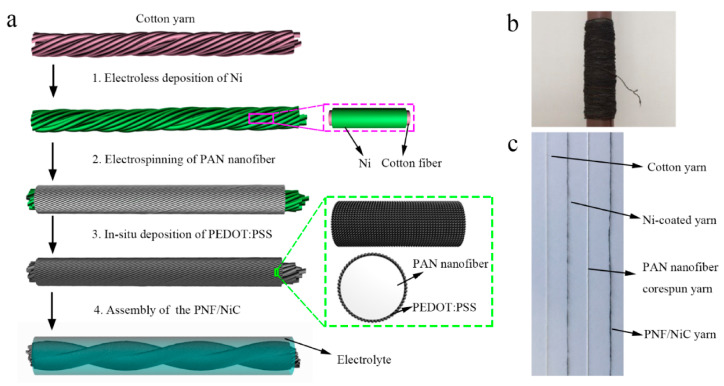
(**a**) Scheme showing fabrication process of PNF/NiC capacitor yarn. (**b**) Image of 500-meter-long Ni-coated CO yarn wound around spinning cone. (**c**) Images of different yarns at various stages of processing [62].

Among all gel electrolytes described in the literature, PVA-based gel electrolytes are among those used for a wider range of applications in combination with different acids or bases. This is justified by most authors as being related to performance issues, since in all cases where more than one gel electrolyte is tested, the best electrochemical results are achieved with PVA. For example, the work of Yang et al. [57] involved YSCs prepared using a PVA/salt gel electrolyte. This method combines Ni_3_(2,3,6,7,10,11-hexahydroxytriphenylene)_2_ (Ni_3_HHTP_2_), a conductive metal–organic framework (MOF), with Au-plated PET/polydopamine yarns to produce all-solid-state symmetrical YSCs. The supercapacitor produced comprises two identical PET/Au/Ni-MOF@carbon yarn electrodes, twisted together while fully coated with the KCl gel electrolyte. The resulting yarns presented high-length capacitance (1.1 × 10^4^ mF/cm, at a current density of 0.1 mA/cm), an energy density of 3.9 × 10^−6^ Wh/cm (at 2.5 × 10^−5^ W/cm), and a power density of 2.5 × 10^−4^ W/cm (at 5.9 × 10^−7^ Wh/cm). No performance loss was observed when a tension up to 42.3% was applied. In the work of Tang et al. [58], ZnSO_4_ was used to coat G/Zn–MnO_2_@stainless-steel CO-blend fibers. This was prepared by the combination of a MnO_2_-based anode and a Zn-based cathode, itself prepared via electrochemical deposition. The battery presented exceptional durability and washing resistance, with 89.66% capacity retention after 30 hammering cycles and 77.97% capacity retention after a 30 min washing cycle. The material also presented a good electrochemical performance, with a 43.53 mAh/g specific capacity and a 51.94 mWh/g energy density. PVA can also be used in combination with inorganic acids such as H_2_SO_4_ or H_3_PO_4_. One example is the work of Uzun et al. [59], who proposed the development of Ti_3_C_2_-coated cellulose yarns via a simple two-step dip-coating process, which was used for the preparation of supercapacitors using a H_2_SO_4_ gel electrolyte. The yarn production was achieved through the dip-coating of cellulose yarns in a homogeneous dispersion of Ti_3_C_2_-based small- and/or large-size flakes. A good MXene cellulose loading capacity was reported (up to 77% wt, around 2.2 mg/cm), resulting in highly conductive yarns (up to 440.3 ± 0.9 S/cm), which could be knitted into full fabrics using different stitch patterns (half-gauge and interlock) making use of industrial knitting machines. In 2020, Du et al. [60] developed a self-powered and self-sensing energy textile system using a H_2_SO_4_ gel electrolyte and cellulose/stainless-steel composite yarns. The yarns were coated with PEDOT via in situ polymerization, followed by sorbitol secondary doping, and the YSCs were produced using two parallel electrodes separated by the gel electrolyte. The fabric prepared by weaving demonstrated a solid areal energy density (1.0 × 10^−5^ Wh/cm^2^) at a power density of 1.7 × 10^−4^ W/cm^2^. Another yarn supercapacitor was produced by Zhang et al. [61], where stainless-steel/CO-blended yarns were coated with polypyrrole (PPy) to produce a highly flexible material. In this work, H_2_SO_4_ was used as the gel electrolyte, and the supercapacitor, produced by electrochemical deposition, presented high areal capacitance (up to 3.4 × 10^2^ mF/cm^2^ at a current density of 0.6 mA/cm^2^) and high cycle stability (approximately 93% capacitance retention after 1000 cycles). As for the H_3_PO_4_ combination, Sun et al. [62] proposed a method to prepare a flexible PEDOT:PSS-poly acrylonitrile/Ni CO (PNF/NiC) capacitor using this specific gel electrolyte (Figure 3). The yarns were first coated by dip-coating in a Ni-containing electroless solution, and then PAN nanofibers were wrapped to produce PAN core-spun yarns. Finally, the yarns were dip-coated in a PEDOT:PSS chloroform solution while dripping on an ethanolic solution of ferric chloride. They were then combined with the H_3_PO_4_ gel electrolyte, resulting in yarns with a high volumetric capacitance (2.6 × 10^4^ mF/cm^3^ at 0.08 A/cm^3^), and good energy (9.56 mWh/cm^3^) and power (830 mW/cm^3^) densities. Carvalho et al. [63] also used H_3_PO_4_ gel electrolytes, paired with in situ regenerated cellulose-based ionic hydrogel, to produce CO/carbon fiber–yarn-based supercapacitors. The yarns were produced via the twisting of CO and carbon fiber yarns. Following this, we coated with electrolytic and gel electrolyte solutions after fixation in an acrylonitrile–butadiene–styrene (ABS) mold. This sustainable approach led to the preparation of stretch-broken carbon fiber yarns, where both the yarns and the cellulose could be recovered after washing with deionized water and drying. The cellulose could then be reused to produce new YSCs without compromising the good electrochemical properties of the material (specific energy density of 1.7 × 10^−8^ Wh/cm^2^, specific power density of 5.3 × 10^−4^ W/cm^2^ at 1 mA/cm^2^, and 92% capacitance retention after 10,000 consecutive cycles). Rani and Sharma [64] also presented a protocol for the preparation of a high-energy-density yarn supercapacitor using H_3_PO_4_ as gel electrolyte. Here, the YSCs were fabricated by the electrophoretic deposition of TiO_2_ nanofibers onto multi-walled carbon nanotube (MWCNTs) and then dip-coated in the gel electrolyte solution. After producing fibers by twisting two electrode yarns together, the resulting device revealed good capacitance (3.7 × 10 mF/cm at 0.1 mA/cm), capacitance retention (90% retention after 10,000 cycles), energy (1.2 × 10^−5^ Wh/cm), and power (3.7 × 10^−4^ W/cm) densities. Another work, presented by Park et al. [65], described the use of KI as a redox mediator to improve the electrochemical performance of gel electrolytes in YSCs. For this, a H_3_PO_4_-KI-PVA gel electrolyte was used to coat carbon fibers, which were then twisted and physically woven together and used to produce the YSCs. The capacitors produced through this methodology presented an enhanced energy storage ability compared to the control, without KI (volumetric capacitance of 363.9 F/L, energy density of 5.1 × 10^−2^ Wh/L). Improved ion diffusion, charge transfer kinetics, and limited moisture conditions were attributed to the presence of KI. In addition, the YSCs also demonstrated a specific capacitance of 13.9 mF (at a current density of 10 µA) when a 7.5 mM solution of KI was used. In 2020, Levitt et al. [66] described the preparation of CO and Nylon^®^-based YSCs using H_3_PO_4_ gel electrolytes in combination with Ti_3_C_2_T_X_ MXene. The yarns were coated with an MXene/water solution using an automated coating setup and then knitted into an interlock rib fashion. This was followed by coating the knitted fabric with the gel electrolyte solution. The resulting YSCs demonstrated high capacitance (up to 7.1 × 10^2^ mF/cm^2^ and 5.2 × 10^2^ mF/cm^2^ at 2 mV/s, in 1 M H_3_PO_4_ and H_3_PO_4_ gel electrolyte, and delivery energy and power densities of 2.5 × 10^−5^ and 4.7 × 10^−7^ Wh/cm^2^, respectively), as well as excellent cycling stability over 10,000 cycles in a gel electrolyte.

One other example can be found in the work of Pal and Subhedar [67], where YSCs were prepared using PEDOT:PSS-coated carbon nanotubes in combination with PVA/H_3_PO_4_ as a gel electrolyte. The authors proposed a biscrolling technique to assemble the hydrophilic carbon nanotubes, where NMP is used to hydrophilize the carbon nanotube forest. In this method, the yarns are assembled by attaching one end of the composite PEDOT:PSS/CNTs-NMP sheet to a motor using sticky double-sided tape, and then twisted at about 2000 turns/min, followed by coating with gel electrolyte. This supercapacitor showed a promising electrochemical performance, having a capacitance of 112.76 F/g, maximum power and energy densities of 9.8 × 10^2^ W/kg (1.2 Wh/kg) and 3.8 Wh/kg (1.5× 10^2^ W/kg), respectively, and cycling stability performance with the minimum loss after 5000 repeated cycles of charge–discharge. Another possible combination of PVA in gel electrolytes is the inorganic base–gel electrolyte. For example, Wang et al. [68] used a KOH gel electrolyte to produce 1D symmetrical yarn-shaped supercapacitors. The NiCo-layer double hydroxide (NiCo-LDH) was grown in situ on the yarns by using a hydrothermal method. Then, PPy was deposited by electrochemical deposition, forming the PPy@NiCo-layered double hydroxide@stainless-steel yarns. Finally, the KOH gel electrolyte was coated after assembling two parallel yarn electrodes on the PET fabric, while a PAN yarn was electrospun on the yarn electrode to prevent short circuiting. The YSCs demonstrated good specific capacitance (1196 F/g at 1 A/g), energy (6.6 × 10^−3^ Wh/cm^3^), and power (1.6 × 10^−1^ mW/cm^3^) densities. In another work from Wang et al. [69], a 3D metallic conductive-coated Au/CO yarn was prepared for the development of flexible energy storage devices using a KOH gel electrolyte. The NiCo_2_S_4_ nanotube array electrodes were prepared by hydrothermal synthesis, followed by the electrodeposition of Ni-CO LDH to produce 3D core–shell structured NiCo_2_S_4_@Ni-Co LDH yarns. The yarns were then used to assemble a two-ply supercapacitor, which presented high capacitance (5.7 × 10^3^ mF/cm) and good areal energy density values (3.5 × 10^−6^ Wh/cm^2^, at a real power density of 1.6 × 10^−4^ Wh/cm^2^), as well as a stable cycling performance, with only a small capacitance retention decrease (9%) after 3000 cycles. Another example of the use of KOH gel electrolyte can be found in the work of Liu et al. [70], where triboelectric nanogenerators (TENGs) and asymmetric YSCs were prepared. In this publication, yarn-based TENGs and YSCs are produced from Ni and Cu film-coated PET yarns, where the negative electrode is prepared by the hydrothermal reaction of self-assembled rGO/CNT and the positive electrode is prepared by electroplating PET yarns with Ni-Co bimetallic oxyhydroxide. The YSCs and TENGs produced from these yarns exhibited high areal energy density (around 7.8 × 10^−5^ Wh/cm^2^), power density (1.4 × 10^4^ W/cm^2^), flexibility (1000 cycles run while bending at 180°), and a stable cycling performance (82.7% capacity retention after 5000 cycles). In 2024, a work by Al-khaykanee et al. [71] used a KOH gel electrolyte to prepare NiMnO3-rGO@CO-Cu YSCs. Here, CO yarns were dip-coated with a graphite and PVDF solution and then woven with the CO wires. After that, the electrodes were prepared by dip-coating with the NiMnO_3_-rGO nanocomposite (prepared by hydrothermal reaction). The YSCs prepared via this procedure revealed good electrochemical performance, with a maximum specific capacitance of 4.0 × 10^2^ mF/cm^2^ (at scan rate of 5 mV/s in a 1 M KOH solution electrolyte), a maximum specific capacitance of 8.3 × 10 mF/cm^2^, and a specific energy density of 1.8 × 10^−5^ Wh/cm^2^ (at a specific power density of 3.8 × 10^−4^ W/cm^2^ in a potential range of 1.7 V). Moreover, the authors also describe how the yarn supercapacitor’s capacitance does not suffer any performance issues when bent, having tested a bending angle up to 210°. A novel battery–capacitor hybrid device was prepared by Li and coworkers [72], using carbon-based yarns (CBYs) in combination with KOH gel electrolytes. Here, an in situ growth procedure was used to prepare CoNi-layered double hydroxide (CoNi-LDH) nanosheets, achieved by hydrothermal reaction at 180 °C. The battery–capacitor apparatus was made by combining two yarns, CoNi-LDH@CBY (cathode) and S-doped carbon nanoparticles on CBY (S-CNP@CBY, anode), coated with a PVA/KOH gel electrolyte. The assembled device presented good electrochemical properties, having a wide voltage operating window of 1.4 V, a large area capacitance (2.3 × 10^2^ mF/cm^2^ at 1.8 × 10^−3^ W/cm^2^), and a high energy density (6.2 × 10^−5^ Wh/cm^2^), while also being stable after 5000 cycles of charge–discharge. The CoNi-LDH@CBY electrodes, highlighted as a novelty by the authors, also presented a large specific surface area (655 m^2^/g) and abundant active sites, contributing to an improved electrochemical performance. Another example of NiCO-LDH-based electrodes that are used in combination with PVA/KOH gel electrolytes to assemble YSCs can be found in the work of Yu et al. [73]. Here, CO yarns were coated with CNT, and were then dip-coated in a solution of (Co(NO_3_)_2_⋅6H_2_O, Ni(NO_3_)_2_⋅6 H_2_O and urea, while we used variable amounts of NH_4_F to tune the NiCo-LDHs morphology into either nanoneedles, nanosheets, needle–sheet composites, or nanoflowers. Then, the supercapacitor is assembled by combining two yarns and either dip-coating them in the gel electrolyte or by placing them in a PET substrate and subsequently coating them with PVA/KOH. The devices prepared in this study presented good areal capacitance (1.2 × 10^2^ mF/cm^2^), good current density (0.2 mA/cm^2^), and high energy density (3.9 × 10^−5^ Wh/cm^2^ at a current density of 0.2 mA/cm^2^), with the best results being achieved with the nanoneedle-shaped NiCO-LDHs. Ahn et al. [74] also developed a yarn supercapacitor using PVA/KOH and PVA/Na_2_SO_4_ gel electrolytes. In their work, the authors prepared transition metal oxide (TMO)-Ni-TMO trilayer nanoribbon yarns by nanoimprinting the TMO-Ni-TMO sequentially in the mold. Then, after the delamination of the nanoribbon array, the yarn was produced by twisting. Finally, the YSCs were produced using two TMO yarns or one TMO yarn and a graphene fiber coated with gel electrolyte. Among the YSCs prepared using different TMOs, the most promising results were obtained with the graphene–CoNixOy@Ni (G-CNO) YSCs, having maximum energy and power densities of 7.6 × 10^−2^ and 2.4 × 10^−1^ Wh/cm^3^, respectively, and displaying 94.2% retention of the initial capacitance after 10,000 cycles. In addition, good flexibility, reported as a 0–0.9% resistance change, was observed at a curvature of 5.21 1/cm; we also saw a stable electrical resistance with <1.00% variations after 1000 bending cycles. Li et al. [75] achieved a combination of different gel electrolytes in the preparation of textile sensor devices. In this work, PVA/H_2_SO_4_, PVA/KOH, and PAAm-NaCl gel electrolytes were used to coat stretchable piezoionic MWCNT-based yarns, which were prepared by simple dip-coating or dip-coating followed by UV curing (for PAAm-NaCl gel electrolyte). The yarns were previously produced via floating catalyst chemical vapor deposition, followed by twisting. The yarns were able to generate sensitive voltage signals (4–15 mV), with only a small noise percentage (0.024 mV) between their endings and asymmetrically stretching, and they also showed the high sensitivity of voltage amplitude to tensile stretches in a wide range of frequencies (0.1–10 Hz) and strains (1–80%). It is suggested that piezoionic activity is derived from so-called “ion-squeezing”, which allows for a current to be generated along the conductive yarn without an external electrical pulse being applied, which is optimal for the application of this material as a smart textile and wearable sensor, as well as for artificial muscle feedback. Another example was presented by Huang et al. [76], where the PVA/LiCl gel electrolyte was used to prepare YSCs via an innovative procedure called additive functionalization and embroidery manufacturing (AFEM). This procedure starts with the polymer-assisted metal deposition (PAMD) of Ni onto CO, followed by the electrochemical deposition of rGO in the yarns. Then, these yarns are embroidered into textile substrates in a variable design fashion. The authors test the yarn supercapacitor capacity when coated with a PVA/LiCl gel electrolyte (voltage window of 0.8 V). The YSCs produced possess good electrical properties, such as an areal capacitance of up to 1.6 × 10 mF/cm^2^ for in-plane MWCNT/Ni-CO@fabric with PVA/LiCl at 0.8V), and a stable cycling performance (97% capacitance retention after 1000 charge/discharge cycles). The authors also highlighted that the AFEM method developed was applicable to several types of yarns, as confirmed by testing using different yarn electrodes produced from MWCNT. CMC-based gel electrolytes were used in the production of a Zn-Ion fiber battery using high-strength cellulose yarns [77]. The cellulose yarns were spun from NaOH/urea aqueous solutions and used for the preparation of the cathode and anode yarns. The cathode was prepared by the in situ polymerization of PANI, followed by coating with a conductive slurry (95 wt.% graphite nanosheets and 5 wt.% cellulose nanofibers), and the anode was prepared by the electrochemical deposition of metallic Zn onto cellulose yarns previously coated with the conductive slurry. The battery was produced by intertwining both electrodes, in combination with a cellulose-based gel electrolyte produced by CMC swelling with an aqueous electrolyte (2 M ZnCl_2_-3 M NH_4_Cl). The Zn-ion battery was electrochemically characterized, where energy densities of 153.2 and 6.1 × 10 Wh/Kg were measured at power densities of 0.16 and 6.5 × 10^3^ W/kg, respectively. Good cyclic stability was also achieved, with the specific capacity maintained at 109.7 mAh/g after 1000 charge/discharge cycles at a current density of 5 A/g.

Similarly to previously reported results about fiber-based materials, the development of yarn-based conductive or energy storage materials often relies on PVA-based gel electrolytes, which are commonly combined with acids and salts (Table 2). Dip-coating and in situ polymerization are widely used techniques for incorporating gel electrolytes into yarn-based conductive or storage materials. These methods enable uniform and conformal coating along the fiber surface, ensuring strong adhesion and intimate contact between the electrolyte and the yarn substrate. This is essential for preserving mechanical flexibility and maintaining stable electrochemical performance under mechanical deformations such as bending, twisting, or stretching. Additionally, both methods are compatible with a wide range of fiber types and can accommodate large-scale or continuous processing. Their simplicity, low cost, and scalability make them particularly suitable for the development of wearable and flexible energy storage devices.

### 2.3. Woven Fabrics

**Figure 4:**
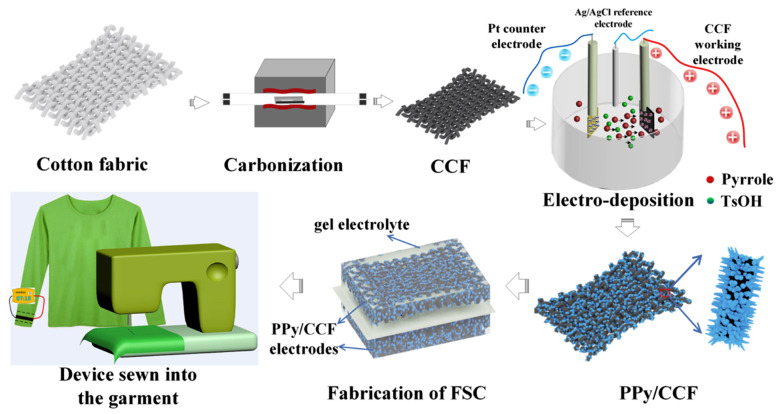
Process flowchart illustrating fabrication of electrode materials and all-solid-state supercapacitor [78].

Woven e-textiles have gained increasing attention due to their remarkable ability to seamlessly integrate electronic functions while maintaining the key advantages of traditional fabrics, which include comfort, a lightweight structure, flexibility, breathability, hydrophilicity, cost-effectiveness, mechanical strength, and softness. Their structured interlaced architecture also enhances durability, wearability, and adaptability for diverse applications [78,79]. Several studies have emerged in the literature that utilize woven structures as substrates for the development of flexible energy storage materials, highlighting their potential for enhanced durability, mechanical stability, and integration into wearable applications (Table 3). CO fabrics, although highly valued, inherently act as electrical insulators. Therefore, their naturally insulating properties require advanced modifications to enhance their suitability for electrical conductivity applications [78]. The carbonization of CO fabric was initially explored to enhance its conductivity and chemical stability (Figure 4). However, this method did not yield satisfactory outcomes for energy storage applications [78]. Recent advanced strategies involve the incorporation of conductive polymers (e.g., polypyrrole (PPy), PANI, and PEDOT) and metallic ions (e.g., copper (Cu), nickel (Ni), cobalt (Co), and silver (Ag)) into untreated fabrics or those subjected to carbonization [79,80,81,82].

**Figure 5:**
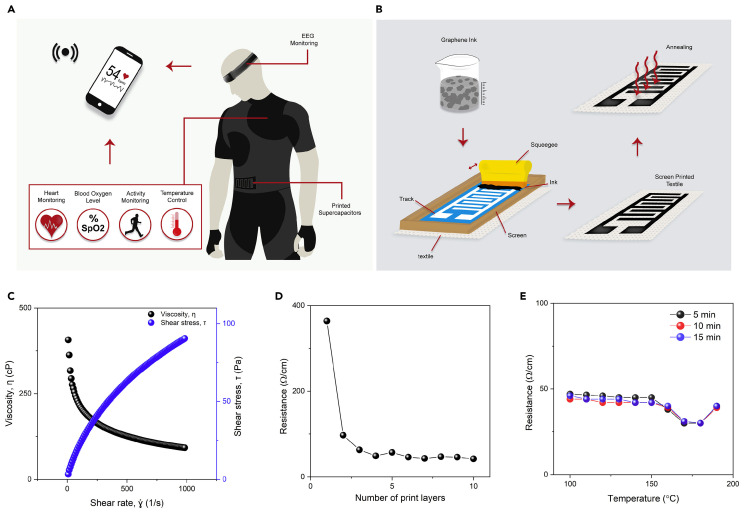
Multifunctional printed wearable e-textiles: (**A**) Illustration highlighting multifunctional features of printed e-textiles. (**B**) Schematic representation of screen-printing technique used to produce graphene-based wearable e-textiles. (**C**) Rheological behavior of graphene ink, showing viscosity and shear stress as functions of shear rate. (**D**) Variation in electrical resistance with increasing print layers on graphene-printed CO fabric. (**E**) Influence of curing time and temperature on electrical resistance of graphene-printed CO fabric [97].

The incorporation of conductive polymers or metallic ions involves the use of gel electrolytes, which are essential for textile supercapacitors. These materials enhance mechanical stability, safety, and adaptability for wearable applications. The flexibility provided by the gel electrolyte allows the system to withstand deformation without losing functionality, ensuring long-term durability. Furthermore, the solid interface between the electrode and gel electrolyte supports efficient ion transport, preserves indispensable moisture levels, and enhances conductivity, resulting in a superior electrochemical performance. PVA is extensively used to produce gel electrolytes for flexible CO-based supercapacitors. Combined with several acids or bases, these electrolytes enhance dielectric reactions and increase ionic conductivity via H^+^ and OH^−^ ions, promoting electrochemical performance [83]. Thus, Sun et al. [84] used PVA/H_2_SO_4_ as gel electrolytes in a supercapacitor produced by spraying a PVA-co-ethylene nanofiber suspension onto CO fabric, followed by electropolymerization with PPy. The nanopores in the PPy increased the electrochemically active spots, enhancing Faradaic reactions. The resulting symmetric supercapacitor achieved high specific capacitance (6.7 × 10^2^ mF/cm^2^), 100% capacitance retention after 10,000 cycles, and efficient Coulombic efficiency (90–100%). Similarly, Wang et al. [80] coated a PPy-based electrode with a PVA/H_2_SO_4_ gel electrolyte. A hydrophobic spray and PU coating enhanced the CO fabric’s hydrophobicity, promoting oxidative polymerization with 5-sulfosalicylate as a dopant. The microsupercapacitor initially lost some capacitance but stabilized, retaining 85% after 3000 cycles, showing good electrochemical stability. An often-used gel electrolyte in flexible textile supercapacitors is PVA/KOH. Wang et al. [85] used this gel electrolyte in CO-based electrodes modified with Ni:Co via electroless plating (cathode) and carbon/Fe_3_C fabric (anode), with silk fabric as the separator. The supercapacitor showed 113.7 C/g at 1 A/g, 80% retention after 4000 cycles, and 4.7 × 10 Wh/kg energy density, indicating potential for high-performance flexible devices. Zhai et al. [86] applied this gel electrolyte by immersing a dyed and carbonized CO fabric electrode, modified with zinc oxide (ZnO) nanoparticles and copper sulfide (CuS) microspheres. These were prepared via atomic layer deposition and a hydrothermal reaction, respectively. The synergistic effect between the electrochemical and pseudocapacitive properties of ZnO and CuS enhanced ion exchange and redox reactions, increasing the substrate’s porosity. The supercapacitor achieved a capacitance of 1.8 × 10^3^ mF/cm^2^ at 2 mA/cm^2^ and 85.2% retention after 5000 cycles, showing excellent performance for supercapacitor applications. Shun et al. [87] demonstrated that carbonized CO fabrics exhibit hydrophobicity (140°), rendering them unsuitable for use as electrodes. However, pre-carbonization dyeing with reactive dye (Procion, 10 wt.%) improved hydrophilicity due to heteroatoms (oxygen (O) and nitrogen (N)), allowing use as electrodes. The pyrolysis of the dye created porous structures with high surface areas, enhancing electrochemical properties and ion deposition. Dyeing increased specific capacitance (1.8 × 10^3^ mF/cm^2^), slightly reduced resistivity (30 Ω/sq), and maintained capacitance retention (~97.4% after 1000 cycles). Using PVA/KOH electrolytes, cellulose separators, and polyethylene terephthalate (PET) coating, the supercapacitor retained 92.2% capacitance after 5000 cycles. Mallick et al. [88] also employed the same gel electrolyte in their study, developing a hybrid supercapacitor through thermal treatment (annealing) with nickel (II) chloride hexahydrate (NiCl_2_·6H_2_O) and sodium tungstate dihydrate (Na_2_WO_4_·2H_2_O). After 20,000 cycles, the capacitance increased by 34% due to nickel(II) hydroxide (Ni(OH)_2_) formation. Furthermore, the one-step Ni:Co deposition method proved cost-effective, achieving a specific capacitance of 60.61 F/g, suitable for supercapacitor material applications. In contrast to the studies mentioned above, Hiller et al. introduced two novel gel electrolyte alternatives: one was inspired by culinary energy drinks [89] and the other was developed using tetraethylammonium tetrafluoroborate (TEABF_4_)/polyacrylamide [83] for supercapacitors produced with CO fabrics functionalized with spray-coated activated carbon. Commercial electrolytes such as Grenade Energy, Shopper Isotonic Drink, and LoSalt^®^ solutions (0.5 and 1 M) were evaluated in the first case. Although energy drinks contained compounds like potassium chloride (KCl), sodium chloride (NaCl), magnesium chloride (MgCl_2_), and dipotassium hydrogen phosphate (K_2_HPO_4_), agar–agar and *k*-carrageenan were added to achieve a gel-like consistency. Among the tested options, the LoSalt^®^ (1 M) electrolyte exhibited a promising electrochemical performance, with a capacitance of 2.3 × 10 mF/cm^2^ at 0.5 mA/cm^2^ [89]. The application of the organic electrolyte TEABF_4_/polyacrylamide exhibited electrochemical properties similar to those of PVA-based formulations, improving durability. This system showed capacitance retention of 48% and a Coulombic efficiency of 88% after two months of aging tests. Although further improvements are needed, the study highlighted practical alternatives to overcome the dehydration limitations of PVA-based electrolytes [83]. Conductive materials often compromise softness and porosity, essential for supercapacitor applications. To overcome this limitation, using gels electrolytes, Wang et al. [79] developed a wearable battery. Their method used CO fabric, activated with palladium (II) chloride (PdCl_2_) and deposited with Co, Cu, Ni, and Ag through electroless deposition. A correlation between conductivity and softness was found in the CO fabrics treated with Co and Ni, revealing conductivities of 1.1 × 10^3^ and 3.3 × 10^3^ S/m and softness values of 8.50 and 8.67 mm, respectively. In contrast, the CO/Cu and CO/Ag fabrics exhibited conductivities of approximately 100 × 10^3^ S/m and softness values exceeding 8.5 mm. The achieved softness addressed common issues in conventional flexible electrodes, enhancing their suitability for wearable battery applications. Chen et al. [90] developed a method using dopamine–cellulose in situ polymerization to overcome low electrode porosity and the application of conductive additives. This was followed by immersion in the GO solution and carbonization. The dopamine multifunctional interface acted as both a binder and conductive additive. After immersion in gel electrolyte, the flexible supercapacitor exhibited nanometric porosity, a high specific surface area (347.6 m^2^/g), and a capacitance of 1.2 × 10^3^ mF/cm^2^, with only a 6% reduction after 4000 cycles. Moreover, Liang et al. [91] selected PPy as the conducting polymer due to its electrochemical properties and high conductivity. GO was applied via vacuum filtration, followed by the in situ ultraviolet (UV) polymerization of PPy and Ag^+^. The π–π interactions between GO and PPy and Ag ions improved electrochemical performance (1.7 × 10^3^ mF/cm^2^ at 0.5 mA/cm^2^, 90.5% retention after 10,000 cycles). UV polymerization generated PPy/Ag nanoparticles, enhancing ion transport and redox reactions (Ag/Ag^+^), with a synergistic effect between PPy, Ag, and GO. Hydrothermal and plasma treatments were evaluated to increase the carbonized CO’s electrochemical properties, overcoming this limitation. Via ring annealing, carbonized CO fabric formed an electrically conductive substrate. After plasma treatment with O_2_ and a hydrothermal reaction with vanadium dioxide (VO_2_) nanosheets, the VO_2_/CO electrode was created, containing ~0.96 mg/cm^2^ of VO_2_. This composite was applied in (i) a flexible piezoresistive sensor with two VO_2_/CO layers separated by a polydimethylsiloxane (PDMS) elastomer substrate and in (ii) quasi-solid Zn-ion batteries with a VO_2_/CO cathode and Zn/CNT film anode, both in the PVA/Zn trifluoromethanesulfonate (PVA/Zn(OTf)_2_) gel electrolyte. The sensor showed 95.8 Ω/sq conductivity, high compression sensitivity (7.12 kPa at 0–2 kPa), a fast response (12/8 ms), and superior 160° flexibility due to the VO_2_ 3D network. Resistance varied with pressure, and energy consumption was low (0.01 V), making the device suitable for lightweight wearable monitoring systems. The battery showed 99.8% Coulombic efficiency, 88.7% capacity retention after 5000 cycles, and a specific capacity of 301.5 mAh/g [92]. The printing process is the most common, straightforward, and cost-effective method of producing flexible CO-based supercapacitors [93]. Keawploy et al. [94] applied conductive ink containing Ag, CNT, and graphene to enhance the conductivity of CO fabric. This enabled the fabrication of an all-solid-state flexible supercapacitor via screen-printing using an Ag/CNT (60/40%) mixture and PVA/KOH as the gel electrolyte. The device achieved a high areal specific capacitance of 6.8 × 10^2^ mF/cm^2^ at a current density of 0.0125 mA/cm^2^, with around 80% capacitance retention after 3000 cycles. Similarly, these authors [95] applied the same modification technique and gel electrolyte, substituting CNT with activated carbon and removing graphene from the ink formulation. These modifications enhanced the specific capacitance to 3.3 × 10^3^ mF/cm^2^ at 5 mV/s and improved the capacitance retention to 130% after 10,000 cycles. The optimization of ink composition is crucial for flexible supercapacitor production. Jiang et al. [96] evaluated four ink formulations based on multi-walled CNT (MWCNT) (5 wt%), thermoplastic PU (2.5, 5, 10, and 15 wt%), and NMP (79.5, 84.5, 89.5, and 92.0 wt%). The optimal formulation contained 5 wt.% thermoplastic PU and 89 wt.% NMP, which produced a porous microstructure, improving electrolyte ion adsorption and increasing capacitance. The supercapacitor achieved a specific capacitance of 26.4 F/g and 1.4 × 10 mF/cm^2^ at 10 mV/s, with stability under a deformation of 20%/s. The same research group [93] also investigated the impact of the structural type used, comparing all-in-one and sandwiched fabric-based supercapacitors. The all-in-one structure exhibited superior performance, achieving a specific capacitance of 4.2 mF/cm^2^, high flexibility, approximately 97.4% capacitance retention after 1000 cycles, and good electrochemical stability, offering easier integration than the typically preferred sandwiched design. Furthermore, washing durability presents a challenge for wearable supercapacitors. Islam et al. [97] developed a solid-state supercapacitor. This was spray-coated with graphene (four layers) for healthcare sensors, using a thin PU encapsulant to protect the conductive polymer (Figure 5). This coating preserved the sensor’s electrochemical properties, maintaining cyclic stability after 10,000 cycles and a capacitance of 3.2 mF/cm^2^ after 10 washing cycles.

**Figure 6:**
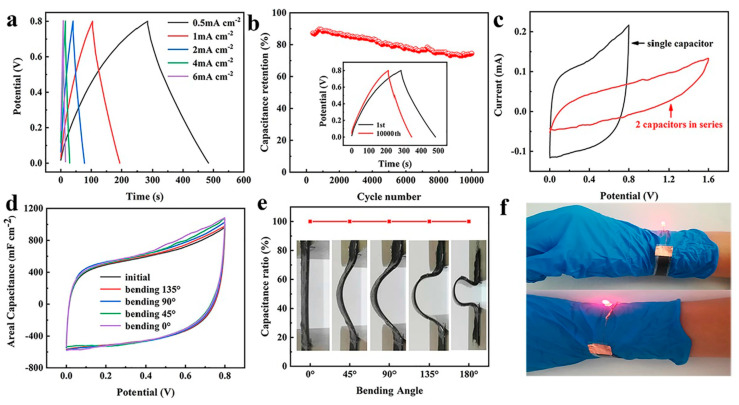
(**a**) Galvanostatic charge–discharge (GCD) test of flexible supercapacitor with 6 wt.% of Ti_3_C_2_T_x_ and corresponding (**b**) cycling performance at current density of 0.5 mA/cm^2^. (**c**) Cyclic voltammetry (CV) curves of single supercapacitor and two supercapacitors connected in series (the single device operates up to 0.8 V), while series configuration reaches 1.6 V. (**d**,**e**) CV curves at different bending angles recorded at a scan rate of 2 mV/s^1^. (**f**) Photograph of flexible assembled device wrapped around wrist, powering red LED [102].

Alongside printing, simple methods, namely dipping and impregnation, can also be applied in the production of textile supercapacitors. Zhou et al. [98] explored an impregnation application method, applying PPy, using iron(III) chloride (FeCl_3_) as an oxidant. They formed FeOx during polymerization and carbonization to enhance ion transfer within the textile structure. The PVA/LiCl supercapacitor achieved 135 F/g capacitance and 88.4% retention after 1000 cycles, indicating potential for energy storage. Integrating multiple conductive polymers offers a viable strategy for improving supercapacitors’ electrochemical efficiency and durability. Thus, Xiaohong et al. [99] developed a flexible symmetric supercapacitor with CO fabric electrodes, functionalized with PEDOT:PSS and in situ polymerized with MXene, graphene nanoscrolls, and PPy. The combination of these conductive polymers—PEDOT:PSS for conductivity and stability, graphene nanoscrolls for mechanical strength, MXene for hydrophilicity, and PPy for flexibility and pseudocapacitance—resulted in a high specific capacitance (4.9 × 10^3^ mF/cm^2^ at 1 mA/cm^2^), with 90% retention after 3000 cycles. The device also exhibited high energy density (3.2 × 10^−4^ Wh/cm^2^) and flexibility, as well as strong waterproof performance (92% capacitance retention after 2 hours in water), demonstrating its suitability for wearable textiles. Costa et al. [100] studied the impact of oxidizing agents on CNT, applied to CO fabrics via the dip–pad–dry method. Nitric acid (HNO_3_), H_2_SO_4_, and an HNO_3_:H_2_SO_4_ (1:3 *v*/*v*) mixture were tested. HNO_3_ increased the number of oxygen-containing groups, enhancing redox reactivity, specific surface area (269 m^2^/g), and pore volume (0.618 cm^3^/g), and thereby improving electrochemical properties. The nitric acid pre-oxidized supercapacitor showed 57, 14, and 106% improvements in capacitance (3.91 F/g), voltage (2.53 V), and energy density (3.5 Wh/kg), respectively, outperforming traditional methods with carbon, Ni, or graphene. Another study by Costa et al. [101] showed that applying MWCNTs to CO fabrics via the dip–pad–dry method, combined with a PVA/H_3_PO_4_ gel electrolyte, resulted in a flexible textile capacitor. High conductivity, thermal, and chemical stability and large surface area ensure excellent electrochemical performance. The superior cyclic stability was attributed to the solid-gel electrolyte, which enhances the capacitor’s durability. Teixeira et al. [1] also evaluated the effect of gel electrolyte composition on a CNT-based supercapacitor’s electrochemical properties (energy storage and fluorescent optical properties). Adding a fluorescent ZnS-Mn pigment enhanced redox reactions, pseudocapacitive charge storage, and UV light response. This resulted in a 4% increase in capacitance (4.37 F/g) and 100% retention after 8000 cycles, showing excellent stability. Moreover, Li et al. [102] developed a flexible supercapacitor using exfoliated and delaminated CO fabric immersed in Ti_3_C_2_Tx (2–8 wt.%), followed by carbonization (800, 1000, and 1200 °C) and immersion in PVA/H_3_PO_4_. The rough CO surface facilitated the adhesion of MXene more than synthetic fabrics. Furthermore, carbonization temperature and MXene concentration influenced electrochemical properties. The optimal performance was achieved with 6 wt.% Ti_3_C_2_T_x_ carbonized at 1000 °C, yielding a specific capacitance of 7.9 × 10^2^ mF/cm^2^ (2 mV/s) and 74% retention after 10,000 cycles. To explore the electrochemical properties of developed materials as flexible wearable energy storage devices, the flexible supercapacitors were assembled by placing two electrodes in parallel and coating them with the PVA/H_3_PO_4_ gel electrolyte. Galvanostatic charge–discharge (GCD) results were displayed near isosceles triangles with no significant voltage drops and IR drops at current densities ranging from 0.5 to 6 mA/cm^2^. The calculated capacitance was found to be up to 5.0 × 10^2^ mF/cm^2^ at 0.5 mA/cm^2^, and 74% capacitance was retained after 10,000 charge and discharge cycles, revealing stable performance at 0.5 mA/cm^2^. In contrast to a single flexible supercapacitor with an operating voltage of 0.8 V, the two flexible supercapacitors devices exhibit a 1.6 V charge and discharge voltage window with similar charge/discharge times. To warrant application, flexible wearable energy storage devices must prove to be flexible enough under different mechanical deformations. Thus, different bending angles from 0° to 180° were tested, showing a slight decrease in capacitance, which was related to the weaker interfacial bonding between the conductive material and textile substrate under these deformation modes. Despite this, wrist-worn supercapacitors can power an light-emitting diode (LED) light source (Figure 6).

Several studies report the influence of gel electrolytes on porosity, facilitating redox reactions and enhancing electrochemical properties. An asymmetric all-solid-state supercapacitor was fabricated by Ramandi et al. [81] by chemically depositing graphene/PANI nanotubes, followed by immersion in NiCo/cetyl trimethyl ammonium bromide and KOH. The NiCo-layered double hydroxide improved electrochemical performance by enhancing redox reactivity and forming a mesoporous structure. Adding potassium persulfate (K_2_S_2_O_8_) to the gel electrolyte promoted ion diffusion, increasing specific capacitance (4.3 × 10^2^ mF/cm^2^ at 0.075 mA/cm^2^) and reducing degradation (15.95% after 10,000 cycles). Liu et al. [103] demonstrated that ion diffusion in the electrolyte, porosity, and surface area are crucial for supercapacitors with strong electrochemical properties. An asymmetrical all-solid-state textile supercapacitor was produced using dopamine hydrochloride, chloroauric acid (HAuCl_4_), a Cu-metal–organic framework (MOF) solution, and a gel electrolyte (PVA/KCl) with PET-sealed electrodes. The Cu-MOF and gold (Au) layers improved ion diffusion and redox reactions, leading to a high specific capacitance (258 F/g at 0.075 mA/cm^2^) and energy density (4.3 × 10^−4^ Wh/cm^2^). Similarly, Wan et al. [104] found that CO fabric improves electrochemical performance due to its bidimensional structure, which enhances ionic conductivity. The flexible supercapacitor, prepared by padding with copper chloride (CuCl_2_), followed by in situ vapor-phase polymerization with PPy in an ice bath and immersion in a gel electrolyte, achieved a specific capacitance retention of 86.5% after 12,000 cycles. Zheng et al. [82] explored vapor-phase polymerization with PEDOT and spray-coated MXene, using PVA/H_2_SO_4_ as the electrolyte. Five layers of PEDOT provided superior performance (1.0 × 10^3^ mF/cm^2^), outperforming values from the literature. Moreover, the synergistic interaction between PEDOT and MXene formed an interconnected network that enhanced electron transport and conductivity. The supercapacitor showed strong electrochemical stability, high thermal performance (193.1 °C at 12 V), effective electromagnetic (EMI) shielding (36.62 dB), and high detection, making it ideal for portable energy storage. Electrodeposition and electrochemical deposition are commonly employed as techniques to enhance the conductivity of CO fabrics through electrochemical reactions. In electrodeposition, the fabric is immersed in an electrolytic solution containing metal ions, and the application of an electric current promotes the deposition of a thin metallic layer onto the insulating substrate [105]. Jin et al. [105] investigated the electrodeposition of CuS nanosheets onto carbonized CO fabrics through two techniques: potential (−1.1 V, 400 s) and galvanostatic (9 mA, 2400 s, 60 °C). The galvanostatic method (g-CuS) provided superior electrochemical performance (4676 vs. 3.5 × 10^3^ mF/cm^2^) due to its higher crystallinity, surface area, and pore size, leading to lower charge transfer resistance (2.2 Ω) and more efficient redox reactions. Upon the immersion of the electrode in a PVA/KOH solution and the formation of a subsequent sandwich structure between cellulose films, polyethylene terephthalate membranes, and Cu wire, a decrease in specific capacity was observed (4675 vs. 1.3 × 10^3^ mF/cm^2^). Despite this decline, the performance remained suitable for wearable energy storage device applications, demonstrating stable cyclic performance.

Conversely, electrochemical deposition is commonly used with conductive polymers, wherein polymer chains are formed through the oxidation and reduction of monomers, which are subsequently deposited onto the electrode surface. Flexible supercapacitor electrodes were produced by Sun et al. [78] using carbonized CO fabric, the in situ electrodeposition of PPy, and immersion in PVA/LiCl. Fabric carbonization improved electron transfer (hydrophobicity), while *p*-toluenesulfonic acid dopant enhanced redox reactions and conductivity. The optimized electrode (PPy/carbonized CO, 1:2) exhibited a high specific capacitance of 3.6 × 10^3^ mF/cm^2^ at 2 mA/cm^2^. PPy deposition improved conductivity, contact area, and hydrophilicity, reducing charge transfer and series resistance. Despite a decrease in capacitance to 5.0 × 10^2^ mF/cm^2^ after immersion in PVA/LiCl and sandwiching with cellulose, the performance remained stable. The electrochemical and comfort properties of CO fabrics, with Au nanoparticles deposited via layer-by-layer, were evaluated. Modification with PDMS resulted in conductive textiles with self-cleaning capabilities (98.88%), durability (60 washing cycles), hydrophobicity (120–140°), breathability, and corrosion resistance. These properties are crucial for supercapacitors, yet further electrochemical studies are needed to assess their impact. The electrodeposition of PANI onto these fabrics produced a solid-state device with an energy capacitance of 3.3 × 10^−5^ Wh/cm^2^ [106]. In contrast, Wang et al. [107] applied electrochemical deposition via CV with Ni onto CO fabrics previously sputtered with Cu and subsequently electroplated. The CV curves revealed that the initial Cu sputtering did not affect the efficiency of the Ni deposition, as Cu was not involved in the redox reactions. However, the Ni-Co-S structure promoted ion transport during the electrochemical process, enhancing electrochemical performance and demonstrating potential for wearable supercapacitor applications. Similarly, Hekmat et al. [108] developed flexible supercapacitors using ultrasonic spray with nickel(II) nitrate (Ni(NO_3_)_2_), followed by in situ chemical synthesis of nickel oxide, the electrochemical deposition of nickel tungstate, and immersion in PVA/KOH. The electrolyte gel provided flexibility and prevented short circuiting, maintaining electrochemical stability even after multiple cycles. This made it a promising solution for wearable devices. Hybrid materials, including MOFs and their derivatives, offer advantages over inorganic particles, enhancing electrochemical energy storage. An asymmetric supercapacitor with a carbonized CO cathode, modified with Zn-N-MOFs-CoNi layered double hydroxides and a Zn-N-modified carbonized CO anode, achieved high energy (4.7 × 10 Wh/kg at 1.6 × 10^3^ W/kg) and power density (2.7 × 10 Wh/kg at 8.0 × 10^3^ W/kg). The high surface area of the layered double hydroxides and the metallic components’ enhanced pseudocapacitance improved electrochemical performance, yielding Coulombic efficiency (>98%), a long cycle life, superior bending resistance, and high specific capacitance (161.25 F/g) [109]. The exceptional electrode performance stems from integrating carbonized CO fabric and Co/zeolitic imidazole framework-67 (MOF-derived), which enhances the supercapacitor’s electrochemical properties. The spinel cobalt oxide–nitrogen-doped structure enables redox processes and charge storage, while CO-derived carbon fibers improve conductivity and flexibility. Nitrogen doping via the zeolitic imidazole framework−67 further enhances performance, achieving 288.62 F/g (0.6 A/g) capacitance, high energy/power density, and 76.4% retention after 2000 cycles [110].

In addition to single-material CO fabrics, various textile substrates have been explored to develop flexible materials with conductive and/or energy storage properties. A notable study was reported by Song et al. [111], in which a H_2_SO_4_-assisted carbonization strategy for synthesizing PANI–carbon–textile electrodes was studied through a facile tandem fabrication procedure. This electrode was prepared using H_2_SO_4_ as an acid dopant and carbonizing assistant. First, a fabric of 95% CO and 5% spandex was immersed in the acidic aniline solution; this was followed by a reaction with ammonium persulfate ((NH_4_)_2_S_2_O_8_). Lastly, the ternary electrode was synthesized by simply drying the fabric in the vacuum-drying oven. The all-solid-state symmetric supercapacitor was assembled by using two PANI–carbon–textile pieces and PVA/H_2_SO_4_ gel as the electrolyte and separator, simultaneously. The optimized electrode, PANI/carbon/textile, achieved an impressive areal specific capacitance of 3.9 × 10^2^ mF/cm and maintained over 70% capacitance retention after 5000 cycles. Furthermore, a symmetrical supercapacitor fabricated from these electrodes delivers a high energy density of 3.6 × 10^−2^ Wh/m at 7.5 × 10^−1^ W/m power density and the capacitance of the device was stable under bending (0–180°) and stretching (up to 50% elongation). In another study, Song et al. [112] developed PANI–graphene–textile electrodes using a straightforward dipping and drying method, followed by in situ polymerization of aniline. This research systematically examines the influence of acidic dopants (HCl, HNO_3_, *D*-tartaric acid or citric acid) on the morphological, structural, and capacitive properties of the electrodes. Among them, the sample prepared with hydrochloric acid (HCl) demonstrates an impressive areal specific capacitance of 1.6 × 10^2^ mF/cm at 1 mA/cm^2^. It also exhibits excellent cycling stability, retaining over 75% capacitance after 10,000 cycles at 10 mA/cm^2^. The all-solid-state supercapacitor assembled from these electrodes achieves a high energy density of 7.6 × 10^−1^ Wh/m^2^ at 1.4 W/m^2^ power density. Additionally, the device maintains stable capacitive performance under bending from 0 to 180°, with 77% retention over 600 bending cycles, confirming its outstanding mechanical flexibility and durability in potential wearable energy storage applications. Pullanchiyodan et al. [113] developed a polyamide fabric-based asymmetric supercapacitor, utilizing metal-coated textiles as both electrodes and current collectors. The device features graphite paste printed on Ag-coated textile (Berlin) as the negative electrode and Ni/Cu/Ag-plated fabric (Nora Dell) as the positive electrode, with a PVA/KCl gel electrolyte ensuring biocompatibility. During electrochemical operation, metal oxides formed on the Nora Dell fabric contribute to pseudocapacitance, while the graphite electrode supports electric double-layer capacitance. The asymmetric supercapacitor exhibited improved performance, achieving an areal capacitance of 3.2 × 10 mF/cm^2^ and an energy density of 2.8 × 10^−6^ Wh/cm^2^ at 25 mV/s. In addition, the device demonstrated strong cyclic stability and self-charging capability when integrated with flexible solar cells, highlighting its potential as a safe and sustainable energy storage solution for wearable electronics. Other substrates have been used for the development of electronic textiles based on gel electrolytes, with CO/spandex mixtures, CO/PET blends, polyamide, PET, silk, aramids, PET/polyamide 6,6, and polypropylene (PP) being some examples. In one study by Pullanchiyodan et al. [114], the researchers investigated the development of fabric-based supercapacitors, using metal-coated textiles (Berlin RS (silk, polyamide), Bremen RS (Parachute silk, polyamide), Nora Dell (polyamide), and Armor FR (PET)) as both active materials and current collectors, combined with a non-toxic PVA/KCl gel electrolyte for wearable energy storage applications. Electrochemical and capacitive performance studies were conducted and compared with a metal-free graphite-printed textile supercapacitor (cellulose-PET fabric) to assess the impact of metal coatings. Graphite electrodes printed on Armor FR (Ni/Cu-coated PET fabric) and Nora Dell (Ni/Cu/Ag-coated polyamide) exhibited considerably enhanced capacitance, achieving 9.9 × 10 mF/cm^2^ and 4.7 × 10 mF/cm^2^, respectively, at 5 mV/s. Notably, this was 24 and 52 times greater than that of the metal-free graphite printed textile. Under the same conditions, the energy densities of Armor FR and Nora Dell supercapacitors reached 8.8 × 10^−6^ Wh/cm^2^ and 4.2 × 10^−6^ Wh/cm^2^. Moreover, the Nora Dell-based supercapacitor demonstrated a stable performance over 5000 charge–discharge cycles, confirming its durability. Furthermore, in vitro cytocompatibility tests with adult human dermal fibroblast cells validated the biocompatibility of the PVA/KCl electrolyte, reinforcing its suitability for wearable applications. In another work, Zhang et al. [115] focused on the development of coplanar supercapacitors on textiles, which offered improved mechanical flexibility and seamless integration with other devices compared to conventional stacked configurations. However, their practical application is often limited by low energy density. To address this, this study proposed an in-plane hybrid supercapacitor on textiles, featuring a specially designed battery-type positive electrode to enhance energy storage performance. This electrode integrates NiCoAl-LDH (for high capacity), Ti_3_C_2_T_x_ MXene (for high conductivity and structural stability), and Ag nanowires, achieving an impressive capacity of 592 C/g (at 1 A/g), along with an excellent rate performance and cycling stability over 10,000 cycles. Utilizing this composite as the positive electrode and activated carbon as the negative electrode, the screen-printed supercapacitor demonstrated a high areal energy density of 22.18 μWh/cm^2^ and a power density of 3.0 × 10^−3^ W/cm^2^, considerably outperforming traditional carbon-based in-plane textile supercapacitors. Furthermore, this device exhibits exceptional bending capability, reinforcing its potential as a printable and efficient power source for flexible and wearable electronics. Kota et al. [116] developed batteries that were useful for body-conformal wearable sensors in a 2D planar textile-based primary silver (I) oxide (Ag_2_O)–Zn battery fabricated using the stencil printing method. The functionalized PET fabric achieved an areal capacity of 0.6 mAh/cm^2^ with an active electrode area of 0.5 cm × 1 cm. The discharge duration of the textile-based Ag_2_O–Zn battery was enhanced by optimizing the PEO-based gel electrolyte. In particular, the authors investigated how the drying time, electrolyte concentration, and PEO weight percentage of the gel electrolyte influence the battery’s discharge capacity. The 5 wt.% PEO gel served as a reliable separator material, exhibiting no signs of delamination after the addition o the KOH electrolyte. To enhance the discharge duration of the textile-based Ag_2_O–Zn battery, the drying time of the PEO-based gel electrolyte was carefully optimized. The optimal drying condition for the 5 wt.% PEO-based gel electrolyte was identified as 10 minutes at 40 °C. Barakzehi et al. [117] published another notable study focused on the development of a textile-based electrode by modifying PET fabric. The fabric was modified with rGO nanosheets and PPy nanospherical particles. These conductive composites enable the fabrication of flexible supercapacitors utilizing a gel electrolyte of PVA/H_2_SO_4_. The optimized device demonstrates an impressive areal capacitance of 2.3 × 10^2^ mF/cm^2^ at 1 mV/s, a volumetric capacitance of 5.5 × 10^3^ mF/cm^3^ at 1.6 mA/cm^3^, an energy density of 1.1 × 10^−5^ Wh/cm^2^, and a power density of 3.0 × 10^−2^ m^2^. Moreover, it retains approximately 76% of its initial capacitance after 6000 cyclic voltametric cycles and exhibits outstanding mechanical stability under bending. In another study [118], the same authors explored the modification of PET fabric with aluminum-based MOFs (MIL-53(Al)) using a layer-by-layer assembly method at room temperature. Subsequently, rGO was deposited via dip-coating in a GO suspension, followed by chemical reduction. Finally, pyrrole was polymerized in situ on the PET/MOF/rGO surface. The resulting composite electrode achieved an impressive areal capacitance of 5.1 × 10^2^ mF/cm^2^ at a scanning rate of 1 mV/s in an aqueous H_2_SO_4_ electrolyte. The optimized electrode was further integrated into a symmetrical solid-state supercapacitor, which delivered a volumetric capacitance of 3.5 × 10^3^ mF/cm^3^, an energy density of 6.4 × 10^−5^ Wh/cm^3^, and a power density of 0.6 × 10^−3^ W /cm^3^. Notably, the device exhibited excellent long-term stability, retaining 85% of its initial capacitance after 1000 cyclic voltametric cycles and maintaining performance even after 12 months of ambient storage. Bhargava et al. studied the electrochemical performance of flexible supercapacitors using three commonly used substrates, namely, commercially available conductive PET fabric (Cu/Ni-PET), carbon cloth, and stainless-steel wire mesh. This study specifically examined how substrate properties, including conductivity, surface morphology, and wettability, affected the performance of PPy–graphene–PPy sandwich structure electrodes. The results revealed that the Cu/Ni-PET-based device exhibited the highest electrochemical performance, achieving an areal capacitance of 6.8 × 10^2^ mF/cm^2^ at a current density of 2 mA/cm^2^ and maintaining 94.2% of its capacitance after 4000 cycles. The superior performance of the Cu/Ni-PET-based supercapacitor was primarily attributed to its high conductivity, favorable wettability, distinctive surface morphology, and excellent flexibility. Afroj et al. [119] applied the pad–dry–cure method to CO-PET (35/65%) fabrics. This group developed a wearable e-textile using graphene ink, an encapsulant, and a PVA/H_2_SO_4_ gel electrolyte. The material achieved 2.7 mF/cm^2^ capacitance and 98% retention after 150 bending cycles at 180°. It also retained conductivity after 10 washes, addressing the durability issues of textile supercapacitors. Several examples of woven textile-based power sources that use PVA/H_3_PO_4_ as gel electrolyte can be found in the literature. In their work, Li et al. [120] proposed a method to produce all-solid-state supercapacitors using silk fabrics and employing PVA/H_3_PO_4_ as the gel electrolyte. This procedure proposes the use of silk fabric, carbonized (CSF) under a nitrogen atmosphere at 900 °C, which is then coated with PPy via potentiostatic electrodeposition. The supercapacitor is assembled using a 2-electrode system with a cellulose separator sandwich and sealed with a PET film. The resulting composite has a mass loading of 16.02 mg/cm^2^ (CSF/PPy), as well as good areal capacitance (4.0 × 10^3^ mF/cm^2^ at 2 mA/cm^2^) and cycling stability (88.6% capacitance retention after 1500 cycles). Regarding the supercapacitor produced, outstanding areal specific (6.7 × 10^2^ mF/cm^2^) and volumetric (1.5 × 10^4^ mF/cm^3^ at the current density of 2 mA/cm^2^) capacitances were achieved, as was an energy density of 6.88 mWh/cm^3^ (at a power density of 4.0 × 10^−3^ W/cm^3^). Sim et al. [121] also proposed another method to produce supercapacitors using PVA/H_3_PO_4_ as gel electrolyte. In this work, the authors prepared Nylon^®^/CNT electrodes by dip-coating Nylon^®^ fibers, which were then used to prepare supercapacitors via lamination. The laminates comprised two Nylon^®^/CNT electrodes, separated by a Nylon^®^ sheet and covered with the gel electrolyte, which were in turn outlined by two Nylon^®^/rubber composite sheets. This material showed promising performance, with a maximum capacitance of 117 F/g (at 2 mV/s) and a maximum energy density of 4.0 Wh/kg (at a power density of 3.5 × 10^2^ W/kg), while losing only a fraction of its performance while under strain up to 200%. Another example can be found in the work of Khairi et al. [122], where woven conductive fibers were prepared using silk and different coatings (namely GO, PANI and GO@PANI composites). These were then tested to assess their electrochemical performance. The capacitors were prepared by dip-coating, with the silk fabric being dipped in either the GO or the PANI solution. The PANI solution, used for both the PANI-SL and the GO@PANI–silk materials, comprised the appropriate reagents to synthesize PANI (namely aniline, ammonium persulphate and nitric acid), and not an aqueous PANI suspension. The authors highlight the GO@PANI–silk fibers as a promising combination that can be used in the production of supercapacitors, presenting the highest specific capacitance of all the coated silk electrodes (450 F/g at 10 mV/s). In addition, a capacitance of 71.2 F/g (at a current density of one A/g) was obtained with the symmetric PANI@GO-SL/PVA/PANI@GO–silk capacitor (using PVA/H_3_PO_4_ as gel electrolyte), displaying 87.4% retention at 5000 cycles. The Ragone plot of the symmetric cell, produced using this material also showed the highest energy (2.5 × 10 Wh/kg) and power (8.0 × 10^3^ W/kg) densities. An all-solid-state supercapacitor was constructed by Stempien et al. [123] using two rGO-coated textile layers combined with PVA/H_3_PO_4_. For this study, PP, PAN, and PET were used as textile substrates, which were coated with GO by reactive inkjet printing in parallel with ascorbic acid, which acted as a reducing agent. The coated textile layers were then coated with the gel electrolyte by drop casting; they were then combined in a two-layer system to assemble the SC. Promising results were obtained with PP, having achieved a maximum specific capacitance of 1.3 × 10 mF/cm^2^ (79.9 F/g) at a current density of 0.1 mA/cm^2^, power and energy densities of 4.6 × 10^−3^ W/cm^2^ and 1.2 × 10^−3^ Wh/cm^2^, respectively, and an increased electrochemical stability that allowed the supercapacitor to maintain around 100% of its original capacitance after 5000 cycles. It also demonstrated good and stable behavior when submitted to bending forces. A direct current triboelectric nanogenerator (DC-TENG) and a supercapacitor were also assembled, using polyamide and carbon fibers as textile substrates to harvest biomotion energy from the electrostatic breakdown of clothes [124]. The TENG was prepared by weaving a polyamide warp yarn around the self-designed mode in 20 rows, followed the production of an interwoven weft yarn (polyamide yarn/Ag-coated polyamide yarn). The results showed that a small-sized DC-TENG (1.5 × 3.5 cm) was able to power 416 LEDs, while the larger DC-TENGs (6.8 × 7 cm) were able to achieve an open-circuit voltage of 4500 V, a short-circuit current of 40 μA, and a short-circuit charge transfer of 4.47 μC per motion cycle. A supercapacitor was also assembled by using PEDOT:PSS-coated carbon fibers, coated with PVA/H_3_PO_4_ gel electrolyte and separated by a cellulose sheet, proving to be able to power a hygrothermograph or a calculator to proper function after 1.5 min of charging. Another example of a gel electrolyte that can be used in the production of woven textile-based power sources is PVA/H_2_SO_4_. This type of gel electrolyte is found in the work of Sun et al. [125], where rGO-coated Kevlar^®^-based woven textiles were used to design supercapacitors, using PVA/H_2_SO_4_ as gel electrolytes. In this paper, the authors described the gelation process through which they prepared the rGO@Kevlar^®^ materials, describing the optimization process that allowed them to find the optimal solution for the gelation step. The optimized procedure allowed the preparation of rGO@Kevlar^®^ fibers with 38.1% rGO, possessing a specific strength of 1.6 MPa.m^3^/kg (comparable to the as-received Kevlar^®^, 2.0 MPa.m^3^/kg) and a specific capacitance of 57 F/g. This procedure was then applied to Kevlar^®^ cloth to produce SC, starting by gelation in an autoclave, in the presence of a GO solution containing NH_4_OH. The supercapacitors were assembled by adding the gel electrolyte to two separate sheets of the cloth prepared under optimal conditions (rGO–Kevlar^®^-90C72h–N_2_H_4_), which were then pressed together under a pressure of apx. 1 MPa for 20 minutes. The supercapacitors were tested for their bending and impact resistance, proving able to withstand impacts of up to 9.1 N and deformations of 90°. In addition to these acid-based gel electrolytes, base-based gel electrolytes can also be used for textile-centered power source applications. One of these cases is presented by Liu et al. [126], where rechargeable textile alkaline Zn microbatteries (micro-AZBs) were assembled by using a multi-component gel electrolyte (KOH, zinc acetate (Zn(Ac)_2_), lithium hydroxide (LiOH), calcium hydroxide (Ca(OH)_2_)) in their composition. The micro-AZBs we assembled by using a Kapton mask as a resistor during Ni electroless deposition and Cu electrodeposition. Then, two interdigitated Cu electrodes were coated with Zn and NiCo BOH nanosheets by electrodeposition, and finally the gel electrolyte solution was drop-coating. The promising results were achieved using an electroplated Zn anode and a Ni0.7Co0.3OOH cathode, which show good energy density (2.6 × 10^2^ Wh/kg), power density (1.0 × 10^4^ W/kg), and stable cycling performance (82.7% for 1500 cycles), as well as satisfactory mechanical reliability (bending, twisting, tailoring, among others). Another possibility is the use of PVA-based gel electrolytes combined with different inorganic or organic salts. In their report, Sundriyal and Bhattacharya [127] stated that an electronic textile can be assembled using PVA/LiCl as the gel electrolyte. Here, the authors reported a device prepared from bamboo fabric, in which the cathode (rGO) and anode (MnO_2_–NiCo_2_O_4_) electrodes were prepared by inkjet printing, using hydrazine as a reducing agent for the former and KMnO_4_ as an oxidant for the latter. The device was then prepared using two layers, i.e., one anode and one cathode, separated by a bamboo fabric sheet and coated with the PVA/LiCl gel electrolyte. The electrochemical tests demonstrated that the MnO_2_–NiCo_2_O_4_//rGO device had a stable performance (within a 0–1.6 V range), as well as a high capacitance (2.1 × 10^3^ mF/cm^2^) and energy density (3.8 × 10^−2^ W/cm^3^), with a maximum power density of 2.7 × 10 W/cm^3^. Additionally, the device also presented a reasonable cycle life, with a 92% capacitance retention after 5000 cycles and low charge transfer resistance (3.2 Ω). Another textile-based supercapacitor was prepared by Pullanchiyodan et al. [128] by using a PVA/KCl gel electrolyte in combination with Ag-coated polyamide Berlin fabric. In this work, two types of flexible supercapacitor were prepared, B-flexible supercapacitors and BGr-flexible supercapacitors, which differed only in the incorporation of a graphite paste (containing also ethyl cellulose and Triton X-100 as dispersant) as a coating, applied to the latter, which acted as the electrode instead of the Berlin fabric. Then, the gel electrolyte was coated on top of either the Berlin fabric (for a B-flexible supercapacitor) or the graphite coating (for a BGr-flexible supercapacitor), after which a separator is laminated on top of the device. In both cases, a Ag wire was applied as an external connection, connected to the fabric via a Ag paste. The electrochemical evaluation of the material demonstrated its potential, possessing an areal capacitance of 1.3 × 10 mF/cm^2^, which was almost 4× higher than the capacitance (3.53 mF/cm^2^) of B-flexible supercapacitor flexible supercapacitors (without the graphite paste). Lastly, an example of a polyvynilidene/bis trifluoromethane sulfonimide lithium salt (PVDS/LiTMDS) gel electrolyte being used in the preparation of textile-based batteries can be found in the work of Khudiyev et al. [129]. In this work, a fiber battery was produced by thermal drawing, combining the anode (LTO), cathode (LFP), gel electrolyte (PVDF/LiTFSI), and conductive polymer (carbon black) in gel forms. A correlation was established between the fiber battery length and its capacity using a 140 m long battery with a discharge capacity and energy of apx. 123 mAh and 217 mWh, respectively. The fibers produced were then woven into the satin fabric using an automated weaving machine to produce a 2D electronic battery. The 2D device was submitted to electrochemical and mechanical tests, demonstrating 96% retention in capacity (after 1000 bending cycles), as well as the ability to sustain a maximum tensile stress of apx. 33 MPa (with a failure strain of apx. 34%). In fact, it could retain its capacity with minimal losses after 10 washing cycles. Table 3 presents strategies for producing supercapacitors using conductive polymers (e.g., PPy, PANI, PEDOT), metallic ions (e.g., Cu, Ni, Co, Ag), and hybrid structures (e.g., MOFs) on untreated or carbonized fabrics, with the dominance of PVA-based gel electrolytes. Methods include electrochemical deposition, electroless deposition, spray-coating, screen-printing, dipping, polymerization, and sputtering. Key performance metrics such as specific capacitance, capacitance retention (max. of 100% after 10,000 cycles), energy density, and power density are used to evaluate electrochemical performance and determine effective designs for energy storage. Despite the focus on electrochemical and comfort properties, research should prioritize biodegradability for a more sustainable, non-toxic alternative to conventional devices. Biocompatible gel electrolytes, such as PVA-based gels, have already shown excellent performance in wearable applications.

Carbon cloth, specifically carbon woven textiles, emerges as a viable type of textile structure due to its durability, flexibility, porosity, and simplicity of integration into various energy storage systems. The usage of this material to produce electrodes (cathodes and anodes) in energy devices is extremely visible. However, despite its growing popularity, there is a major paucity of information regarding the construction parameters of these structures. As a result, many published studies do not provide the full characterization of the structural properties of the carbon fabrics utilized, inhibiting the direct comparison of the influence of structural parameters on device efficiency. According to the scientific literature, carbon textiles are widely applied as structural components in a variety of energy storage devices, including batteries and supercapacitors, as shown in the summary table (Table 4). Researchers justify this fact by citing its three-dimensional structure, which combines carbon’s high conductivity with the ability to provide mechanical support and the necessary porosity to optimize interaction with the electrolyte, resulting in ease of ion and electron transport, making it ideal for improving the performance and durability of these devices. Ping Li et al. [130] experimentally showed the first prototype of a highly flexible quasi-solid-state Zn–polyaniline-*co*-azure C (PANAC) battery at the level of battery applications. Providing a rich protonated nitrogen in the charged state and a special ability of “proton self-supply”, a considerable increase in the electroactivity of the polymer in nearly neutral aqueous electrolytes occurred while permitting rapid electron/ion transport. The experiment’s challenge was to conformally assemble the PANAC copolymer onto a carbon cloth wrapped in porous carbon, which was used as a cathode. The researchers revealed the peculiarity of developing a bicontinuous porous carbon-sheathed carbon cloth (CC-PC) cathode with a hierarchical and porous structure to increase the active polymer load (PANAC) and provide rapid ion and electron transport. Comparing the CC-PC@PANAC cathode to the Pt-plate@PANAC cathode (produced by electropolymerization on a flat platinum (Pt) plate under equivalent conditions), the CV curves of the electrodes (at various scan speeds) show that Pt-plate@PANAC cathode has a much lower capacity and rate performance. This demonstrates the critical role of the CC–PC@PANAC’s hierarchical 3D electrodynamic architecture, which can enhance electron transfer and decrease ion transfer distance while increasing the percentage of near-surface/surface reactions. Applications for this kind of hierarchical architecture in supercapacitors are also being investigated, employing carbon cloth as a support framework. The research of Po-Yuan Cheng et al. [131] studied high-performance flexible gel-type symmetric supercapacitors. The authors fabricated a nitrogen-doped hierarchical porous carbon nanostructure (NHPCN) onto a carbon cloth (CC), utilizing self-organized mesoporous silica spheres as a template. For this effect to be replicated, the porosity properties of the CC support structure proved to be highly significant. Because of this property, mesoporous silica spheres were utilized to form a mold in the original CC support structure, which allowed the formation of a thin hollow hierarchical porous carbon nanostructure with large pore volumes and high specific surface areas. Thus, a layer of N-doped carbon was created by etching away the silica mold and subsequently forming thin layers of carbon (polydopamine carbonization). This layer was able to provide electric double-layer capacitance and pseudocapacitance. This was made possible by the adsorption/desorption of electrolyte ions on the surfaces of the high-specific-surface-area electrodes as well as by surface redox reactions, made possible by their numerous nitrogen-doped active sites. This fact was confirmed in the results by comparing two symmetric supercapacitors, using LiCl/PVA as the electrolyte. The electrodes created without MSS templating exhibited lower energy density, power density, and specific capacitance. Along with functionalization procedures that guaranteed optimal attachment and the interaction of functional particles with carbon structures, the study by Xiao-Man Cao et al. [132] evidenced the need to functionalize electrodes made with the CC support structure to promote improved electrochemical reactions. By using a technique known as “root-etch-wrap,” scientists have shown that it is possible to combine the benefits of a stable structural design with electrodes and components to obtain a synergistic effect. Thus, to produce the electrodes, the authors deposited hollow ZnO spheres onto the activated CC through an in situ growth process. Next, the hollow core and shell structure ZnO@ZIF-8 were synthesized in a controlled manner through a controlled synthesis process of ZnO@ZIF-8-CC, forming a ZIF-8 MOF shell composed of interconnected nanoscale crystals. After that, PANI was electropolymerized on the inner and outer surfaces of the ZnO@ZIF-8 structure, forming a uniform and dense layer that resulted in the overall PANI/ZnO@ZIF-8-CC structure. Analyzing the various structures produced by each functionalization step, this research revealed that the final structure had a lower porosity but a higher specific capacitance level, which was explained by the ZnO, ZIF-8, and PANI particles. This indicates that the PANI/ZnO@ZIF-8-CC structure has a substantially lower equivalent series resistance (Rs = 1.22 Ω) than ZnO@ZIF-8-CC (Rs = 6.92 Ω). These features make these structures effective for building energy storage devices. In this study, a high energy density of 1.4 × 10^−4^–8.9 × 10^−5^ Wh/cm^3^ (at a power density of 1.4–2.4 × 10 W/cm^3^) and good long-term cycling capacity (87% after 10,000 cycles at 5 mA/cm^2^) were demonstrated after a supercapacitor system was assembled using a PVA/KCl gel electrolyte intercalated between the PANI/ZnO@ZIF-8-CC electrodes. Functionalizing CC-based electrodes boosts energy storage efficiency by enhancing conductivity, surface area, and porosity. However, the study conducted by Xia et al. [133] highlights the importance of not only optimizing the structures that compose the electrodes of electronic systems but also exploring new electrolyte formulations to enhance the redox reactions essential for improving the electrochemical performance of the supercapacitors produced. In order to enhance electrical conductivity and provide pseudocapacitive qualities, the researchers first created electrodes by depositing them onto a CC structure using a PANI/CNTs core–shell structure via a layer-by-layer growth process. Therefore, in contrast to the CC hierarchical structures, the H_2_SO_4_/0.02M Fe^3+^/Fe^2+^ electrolyte gel was created by adding iron(II) sulfate heptahydrate (FeSO_4_·7H_2_O) and iron(III) sulfate (Fe_2_(SO_4_)_3_) to an aqueous solution of H_2_SO_4_-PVA to enhance the redox reactions between the electrolyte and the electrodes. Comparing electrochemical properties using a H_2_SO_4_ electrolyte, it was possible to verify the presence of new redox peaks centered between 0.30 and 0.55 V, resulting in a higher current density. Also, the increase in the capacitance properties of the electrodes favored by the Fe^3+^/Fe^2+^ ion pair was very notable, proving that interventions at the level of the electrolyte can directly influence the properties of the materials that functionalize the CC structures. In another study, led by Han and coworkers [134], sodium perchlorate (NaClO_4_)/PVA gel electrolyte was combined with CC to achieve a biologically compatible quasi-solid-state supercapacitor. In the study, the MnO_2_ nanowires/CC electrodes were prepared by dip-coating the carbon cloth in a KMnO_4_ solution, after which the mixture was submitted to hydrothermal treatment at 180 °C in an autoclave for variable periods of time. Then, the fibers were disintegrated by a hydrothermal reaction and used in combination with activated carbon fibers and a NaClO_4_/PVA gel electrolyte previously prepared by the freeze–thaw method to produce the flexible supercapacitors. The devices assembled through this procedure presented good capacitance retention, with only 19% performance loss after 25,000 cycles while exposed to external environmental conditions. Additionally, the device also presented stable and consistent charge storage, being able to operate under a −40 to 40 °C range, with only a 40% loss in specific capacitance after 7000 cycles. This brings novelty to the field, since it constitutes a device that, mainly due to the hydrogel used, affords good water retention and frost resistance, while maintaining good electrochemical performance. Mohd Shoeb et al. [135] also proposed the use of woven carbon fibers (WCFs) to assemble high-performance supercapacitors, with the aid of PVA/Na_2_SO_4_ and PVA/EMIBF_4_ gel electrolytes. This study presents a recent methodology to assemble the supercapacitor, namely Vacuum-Assisted Resin Transfer Molding, where the device is assembled in a chamber containing the electrodes and the gel electrolyte (PVA/Na_2_SO_4_), to which a PET resin containing an additional gel electrolyte (PVA/EMIBF_4_) is then added, with both functioning as electrode separators. After adding a curing agent, methyl ethyl ketone peroxide, while decreasing pressure, the chamber is then enclosed to ensure constant temperature (room temperature, in this case). The electrodes used in this study were prepared by a hydrothermal reaction at 180 °C for 12h, using a solution of samarium (III) nitrate hexahydrate (Sm(NO_3_)_3_·6H_2_O) and ammonium metavanadate (NH_4_VO_3_). Then, the carbon nanotube–SmVO_4_, MoS_2_ nanocomposites was also developed via hydrothermal treatment at 180 °C, for 48h, using the SmVO_4_-MoS_2_ matrix previously prepared. The authors report an excellent electrochemical performance, with a specific capacitance of 1.0 × 10^3^ mF/cm^2^ being achieved at a current density of 2.187 mA/cm^2^ for Sm-Mo-C5 in a three-electrode system. The authors highlight that performance improvement is related to a synergistic effect between the SmVO4,MoS2, and CNTs in the composite, which significantly enhances conductivity and active site availability. Regarding the supercapacitor, the scientists highlight its exceptional performance, with the setup achieving a specific capacitance of 2.9 × 10^5^ mF/cm^2^ at a current density of 2 A/cm^2^, 72.5% capacitance retention after 50,000 charge–discharge cycles, and a maximum energy density of 8.9 × 10 Wh/Kg at a power density of 1.0 × 10^3^ W/Kg. This brings a new approach to the field of automotive and aerospatial engineering, as this device can operate in conditions of controlled space and weight while maintaining efficiency and durability. Lin et al. [136] proposed and studied another intervention at the electrolyte layer level, aiming to develop an electrolyte that ensures strong electrochemical interactions with electrodes fabricated on a CC support substrate functionalized through the electrodeposition of PANI nanowires. Additionally, this electrolyte was designed to incorporate self-healing capabilities, allowing it to recover after deformations in the overall electronic system. The self-healing hydrogel electrolyte was developed from an Fe dual physically cross-linked polyelectrolyte, with acrylic acid (AA) as a hydrophilic monomer and stearyl methacrylate (C18) as a hydrophobic monomer in an aqueous solution of iron nitrate, along with the cationic surfactant cetyltrimethylammonium bromide (CTAB). The resulting hydrogel, containing a mild quantity of H_2_SO_4_, exhibited high ionic conductivity (>30 mS/cm), excellent self-healing efficiency, and high extensibility. The copolymerization was carried out, simply by providing an electrolyte with mobile protons that generated capacitance equivalent to that of conventional systems, such as PVA/H_2_SO_4_. With a self-repair capacity of roughly 86% following the seventh healing cycle, the scientists demonstrated that self-repairable supercapacitors demonstrated exceptional electrochemical performance. Additionally, it demonstrated outstanding cycle stability and good resistance to aging, with a minor decline in performance under environmental conditions after a month. To summarize, CC structures are highly valued because of their intrinsic material qualities, which include electrical and mechanical capabilities as well as porosity structural features. As a result, the necessity of the activation/functionalization of CC structures is the focus of ongoing research and experimentation aimed at improving specific features for energy devices. Concurrently, the benefits of operation are demonstrated, not only at the structural level (with usually hierarchical structures), but also through the enhancement of the electrolytes used to increase electrochemical interactions and synergistic relationships, thereby improving the efficiencies of the energy devices developed.

### 2.4. Knitted Fabrics

**Figure 7:**
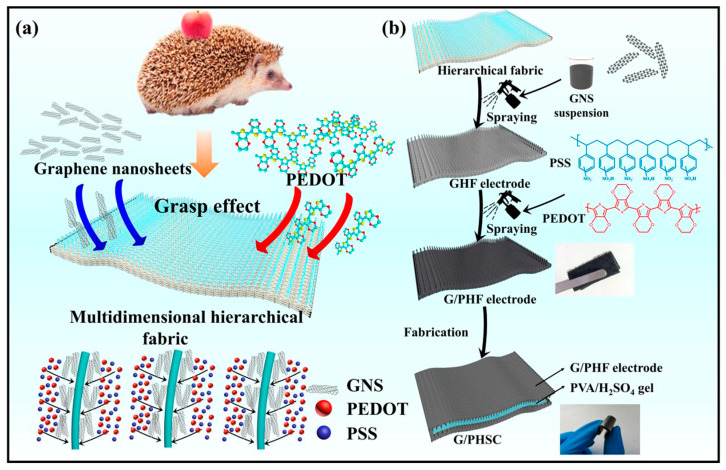
(**a**) Scheme showing deposition of active materials onto hierarchical fabric structure. (**b**) Illustration outlining preparation of graphene nanosheets (GNS)/PEDOT-coated fabric electrodes and assembly process of fabric-based supercapacitors [139].

The scientific literature highlights the advancement of electronics as a key factor in the progress of flexible energy devices. In this context, the use of knitted fabrics has emerged as a promising approach for these devices (Table 5). These structures are in high demand and have been extensively researched due to their versatility. They are particularly valued for their ability to integrate with wearable technologies and other flexible devices. When functionalized, these textile substrates enable devices to exhibit high power density, long lifespan, fast charge and discharge capabilities, and safety. They also offer high flexibility and adaptability in terms of application. Thus, in this research field, there is growing interest in the use of textile substrates, particularly in knitted fabric-type structures, which, as justified in material selection for the development of flexible electronic devices, are typically chosen for being highly stretchable, flexible, and breathable, as well as comfortable for use [137,138]. Studies such as those conducted by Zengqing Li et al. [139] demonstrated that the surface properties of knitted fabrics are crucial for improving the efficiency of these energy storage devices. In this work, bionic fiber microarrays could use the active materials such as graphene nanosheets (GNS) or PEDOT:PSS) as hedgehog spines. The suspension comprising GNS and PEDOT:PSS was successively deposited onto the hierarchical fabric through the spraying strategy. The knitted fabric structure produced played a pivotal role and provides an advantage due to its versatility in the possible structures that can be produced. The study revealed that the three-dimensional configuration of the knitted fabric allows for the optimization of energy storage and charging properties, providing a larger contact area between the electrode and the electrolyte. Thus, a multidimensional knitted fabric with a microarray structure of bionic fibers was developed, specifically for applications in supercapacitors. Due to the “capture effect” of the bionic microarrays, the textile electrode can accommodate more electrochemically active materials, while the significantly high-accessibility interfacial area allows the active materials to be fully exposed to the electrolyte, significantly facilitating ion diffusion during the electrochemical process. In this way, the knitted fabric structure not only increases the storage capacity but also improves the performance and efficiency of energy storage devices such as supercapacitors (Figure 7). In this way, the scientific research conducted by Chuanli Sue et al. [140] demonstrated that the use of a novel diamond-shaped stainless-steel mesh (SSM) in an adapted knitting process, combined with a coating of a three-dimensional graphene network, enabled the construction of a textile substrate with a configuration that considerably increased the available surface area. This allows for the deposition of more conductive material, which can improve the charge capacity and overall efficiency of these devices.

Another advantage of using these textile structures is the ability to integrate multiple electronic devices, referred to as all-in-one textiles. In terms of wearable applications, these not only prioritize the intrinsic properties of knitted fabric structures but also indicate the growing demand for the development of self-sufficient technical structures. They can be manufactured using existing textile substrate production lines, ensuring commercial viability [141,142]. This fact was proven by the construction of a self-sustaining coplanar and stretchable energy textile (SCPT) by Zifeng Cong et al. [141]. These knitted fabrics integrated TENG through a method similar to resistance dyeing, with applications in energy harvesting devices, microsupercapacitors (MSCs), and energy storage devices. In this article, the researchers demonstrated the functionalization of knitted fabric by printing with conductive material on the textile structure. The authors tested rGO in order to produce textile electrodes. Regarding the reproducibility of the MSCs, the application of the PVA/LiCl electrolyte coating by immersion was necessary to achieve proper functionality and electrochemical reactions. In the study performed by Heun Park et al. [142], the authors produced a high-performance, dynamically stretchable supercapacitor to power an integrated sensor of the same structure in order to detect various biosignals using spray techniques. In this context, the supercapacitor and the deformation sensor were fabricated in two distinct stretching directions of the knitted fabric. MWCNT and molybdenum trioxide (MoO_3_) nanowires (NWs) were manufactured for both the stretchable supercapacitor electrodes and the deformation sensor. These pseudocapacitive materials presented high sensitivity, stable performance, and fast response times. PVA-based electrolytes are also commonly applied in knitted fabrics due to their elastomeric hydrogel properties. They can be used as adhesives to improve the bonding forces at the interfaces of the electrodes, transforming into an elastomeric composite film, which adapts to tensile deformation without mechanical degradation [141]. This was also corroborated by the study conducted by Bo Wang et al. [143], who investigated the capacitance stability of electrodes with 0 and 40% strain, initially using a liquid electrolyte (NaCl) and a gel-type electrolyte (PVA/H_2_SO_4_). Although capacitance values were lower with the gel-type electrolyte, justifiable by the relatively poorer diffusion process of the PVA/H_2_SO_4_ gel compared to the liquid electrolyte, the adjusted capacitance values of the electrodes (15.2, 41.2, and 95.5 mF for 0, 20, and 40% strain) showed a higher increase rate due to the deeper diffusion process of the gel. Ongoing research in this field aims not only to enhance the properties of materials but also to ensure that the transition from rigid to flexible devices occurs efficiently, presenting the use of knitted fabric structures for these purposes as a viable solution. In more recent studies, Yavuz et al. [144] highlighted the benefits of electrodepositing a manganese–copper (Mn-Cu) alloy onto a knitted fabric substrate made of graphite filaments. This approach, along with the construction of electrodes for the development of flexible energy storage devices, demonstrated the advantage of combining two compounds that are typically used individually in the production of electrolytes. Specifically, the mixture of KOH and Na_2_SO_4_ combines the benefits of both electrolytes, yielding the excellent ionic conductivity of KOH and the electrochemical stability of Na_2_SO_4_. This combination results in improved overall performance, emphasizing the synergistic effect of the metal alloy in enhancing charge storage capacity.

### 2.5. Non-Wovens

Applications comprising non-woven structures correspond to the least found applications in the literature (Table 6). This may be mainly due to the importance of fiber orientation in conductivity. A positive correlation between fiber orientation and electrical conductivity was observed [145,146]. The differences between oriented and non-oriented fiber reached nearly 2-fold S/cm [146]. However, fiber orientation is not always feasible or simple to achieve, as seen with wet-spinning and electrospinning synthesis processes. Notwithstanding the randomly distributed fibers, wet spinning has been proven to be successfully applied to develop fiber-based supercapacitors, batteries, gel polymer electrodes, and battery separators [147]. Several works can be found in the literature using non-woven and gel electrolytes prepared by wet spinning. Using a two-phase microchannel conical reactor, porous GO was injected and underwent interphase with ethanol, creating a porous GO filament enveloped by ethanol that led to its coagulation. The generated filament was drawn by a rotor in ethanol and acetic acid solution at 60 °C for 8 hours. The obtained filaments were collected through filtration and consolidated using a hot press. Two porous GO non-woven meshes were used to produce a laminated structure, consisting of a sandwich of the two meshes enveloping a PVA/H_2_SO_4_ electrolyte gel. The laminated structure was further consolidated through hot pressing. The obtained non-woven laminated superconductor displayed an impressive performance, with a high capacitance of nearly 1.4 × 10^3^ mF/cm^2^, high energy density of approximately 1.2 × 10^−4^ Wh/cm^2^, and ultrahigh stability within 60,000 cycles. These results were correlated with the high specific surface area of approximately 220 g/m^2^, notwithstanding adequate mechanical properties (1.2 MPa). As an interesting remark, the authors successfully applied the non-woven laminated supercapacitor as a power source for a powered boat, a self-powered fan, and a smartwatch, demonstrating its validity and eclectic applicability [147]. Qiu and coworkers [148] also developed a non-weaving-based supercapacitor through wet spinning in an attempt to develop a wearable supercapacitor with adequate mechanical properties and high energy density. In a MgSO_4_ coagulation bath, microfluidic wet-fusing spinning chemistry was used to produce spin-orientated delaminated Ti_3_C_2_T_x_-MXene graphene-containing quantum dots. After consolidation through filtration, a radical polymerization reaction of PANI was performed on the non-woven surface. The energy density was 21.19 mWh/cm^3^, the power density was 9.8 × 10^−1^ W/cm^3^, and the materials able to withstand 5000 cycles. The high specific surface area, which reached nearly 64 g/m^2^, was also mentioned as a key factor for both ion accumulation and transfer. The developed non-woven supercapacitor was connected to a solar cell for charging and converted the energy to power a wearable health monitor on the wrist and an LED.

**Figure 8:**
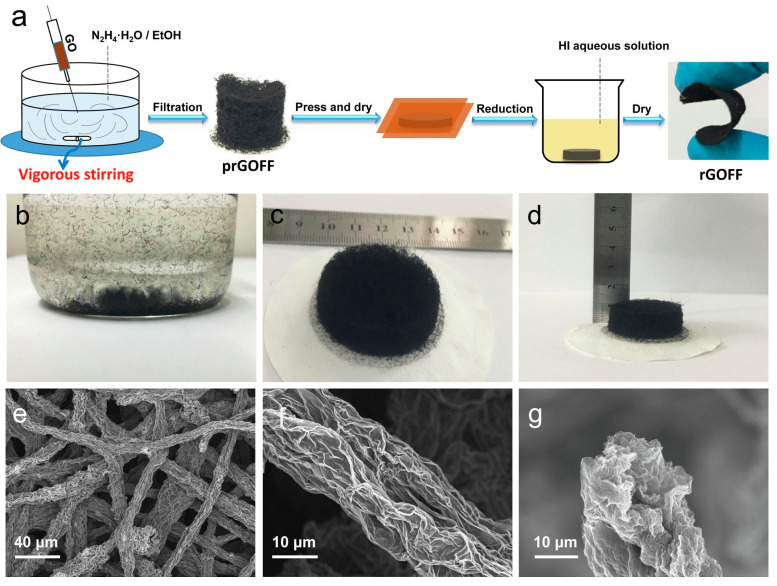
(**a**) Procedure to produce three-dimensional reduced graphene oxide fiber fabric electrodes. (**b**) Digital photo of short partially reduced graphene oxide gel fibers in ethanol. (**c**,**d**) Digital photos of free-standing fabrics. (**e**–**g**) SEM images of reduced graphene oxide fiber fabrics [149].

Another study reported by Shao et al. [149] used a novel coagulation bath fabrication technique to prepare reduced graphene oxide fiber fabrics for areal-energy-dense supercapacitors. Scanning electron microscopy images revealed that the produced fibers possess a three-dimensional, non-woven fabric-like structure composed of overlapping reduced graphene oxide fibers. Each individual fiber presented a diameter of approximately 20 μm, with clearly visible wrinkled microstructures on their surfaces. The non-woven supercapacitor CV curve was approximately rectangular at 300 mV/s, denoting a high-rate performance. For practical use, flexible and symmetrical all-solid-state supercapacitors were constructed by placing the PVA/H_2_SO_4_ gel electrolyte between two layers of reduced graphene oxide fiber fabrics (60 μm, 2.8 mg/cm^2^). The current density was not impaired after bending the non-woven supercapacitor at 90 and 180°. It exhibited a specific gravimetric capacitance of 285 F/g at 0.1 A/g and 220 F/g at 1 A/g. Its area capacitance reached 8.2 × 10^2^ mF/cm^2^ at 1 mA/cm^2^ and 3.2 × 10^2^ mF/cm^2^ at 20 mA/cm^2^. Finally, its gravimetric energy density ranged from 28 μWh/cm^2^ to 1.1 × 10^−5^ Wh/cm^2^, and could provide energy for 3 minutes to a 3 V LED (Figure 8).

Electrospinning is a popular strategy for the development of both conductive non-wovens and separation membranes. In one study from Liu et al. [150], poly(ε-caprolactone) was dissolved in poly-ε-caprolactone (PCL) hexafluoroisopropanol. Afterwards, silicon dioxide nanoparticles were added in different ratios. The electrospinning setup encompassed a distance between the needle and the collector of 12 cm, a flow rate of 1 mL/h, and a voltage of 18 kV. Then, the electrospun mat was dipped in 1 M of lithium perchlorate. Subsequently, electrochromic devices were produced by PEDOT:PSS screen-printing on indium tin oxide-coated PET electrodes. The porosity, electrolyte uptake, and mechanical properties peaked at 3 wt.% of silicone dioxide (SiO_2_) nanoparticles. The electrospun mats exhibited negligible losses of efficiency after 100 cycles at different bending angles (20 and 40°). They exhibited good conductivity 5.2 mS/cm, and an impressive electrolyte uptake (higher than 800%). Another example was the study developed by Singh et al. [151], where polyvinylidene fluoride was dissolved in a mixture of *N,N*–dimethylformamide and acetone (85:15 *v*/*v*). The electrospinning process unfolded at a flow rate of 0.6 mL/h, 16 kV, and the distance between the needle and the rotating drum (at the speed of 1400 rpm) was 15 cm. After a drying step, the electrospun membrane was dipped in the electrolyte magnesium perchlorate (MgClO_4_) at 0.3 M, achieving the preparation of a gel polymer electrolyte comprising fibers averaging 335 nm in size. An ionic conductivity of nearly 1 mS/cm was observed, with a voltage range of up to 5.0 V. However, the thermostability of the gel polymer electrolyte was relatively poor at temperatures higher than 90 °C, which can considerably limit their application.

Dipping or injection was the last strategy employed for the development of e-textiles with gel electrolytes using non-woven structures. Yang et al. [152] prepared a high-performance flexible gel electrolyte by adding poly(ethylene oxide) powder to a mixture containing graphene aqueous solution, *N,N*-Dimethylformamide and poly(vinylidene fluoride-hexafluoro pentaene (PVDF)) in acetone, and a brown slurry was obtained. An undisclosed non-woven was dipped in the slurry and dried. Once dried, it was immersed in a liquid electrolyte composed of bis(trifluoromethanesulfonyl)imide in tetraethylene glycol dimethyl ether, thus producing a gel polymer electrolyte. The gel polymer electrolyte exhibited a hydrophobic nature up to 15 minutes and a battery was subsequently assembled using a disk made of gel polymer electrolyte, coated with a conductivity of nearly 0.3 × 10^−3^ S/cm. The poly(ethylene oxide) formed hydrogen bonds with graphene oxide, generating high interface stability. This allowed the battery to power an LED panel for over 750 h without observable dendrite formation at 0.5 mA/cm^2^. Furthermore, it was stable for 300 cycles of charge–discharge using 1 mA/cm^2^. In another work [153], pure cellulose non-woven fabric with a thickness of 0.38 mm and a weight per area of 48 g/m^2^ was immersed in a graphene suspension. Afterwards, it was dried and immersed in an aqueous solution of potassium permanganate. After a drying step, ethyl alcohol was dropwise added onto the textile substrate. A solid-state supercapacitor was assembled using two non-woven substrates that sandwiched an H_2_SO_4_-PVA gel. Finally, both outer layers of the textile substrates were coated with carbon paper as electrodes. A supercapacitor was obtained, displaying a high specific capacitance of approximately 1.4 × 10^2^ mF/cm^2^ at 0.5 mA/cm^2^ and exhibiting relevant flexibility and cycle stability up to 2500 cycles. Interestingly, the same methodology was applied to a woven textile of pure cellulose, exhibiting a thickness of 0.2 mm and a weight per area of 130 g/m^2^. However, it exhibited poorer performance in comparison to the non-woven. A non-woven textile, of an undisclosed nature, was used as a scaffold for in situ polymerization by Shao et al. [154]. Three main components were mixed: poly(ethylene glycol) diacrylate (PEGDA), ethoxylated trimethylolpropane triacrylate (ETPTA), and liquid electrolyte. The liquid electrolyte encompassed ethylene carbonate, diethyl carbonatem and lithium hexafluorophosphate (LiPF_6_). Azobisisobutyronitrile (AIBN) was added as the initiator, and the solution was injected into a micro- to nanofiber-sized non-woven. After complete cross-linking, a gel polymer was achieved that was subsequently assembled into a battery, stacking a lithium anode with lithium hexafluorophosphate as the cathode. The battery was able to withstand 200 cycles without considerable Coulombic efficiency loss and exhibited an ionic conductivity of approximately 9 × 10^−4^ S/cm. The authors used the same combination of chemicals to produce a gel between wafers of the same cathode and anode, and differences were observed. The latter battery exhibited a poorer performance in all performed characterizations. This was most likely due to the nano- and microporous architecture of the non-woven; however, this feature was not described or explored. Nevertheless, the battery successfully powered an LED setup.

## 3. Perspectives

The growing interest in sustainable energy solutions has driven a significant shift toward the development of ecofriendly and scalable gel polymer electrolytes for use in flexible and wearable energy storage devices. Electrolytes play a pivotal role in electrochemical systems by transporting ions and determining the electrochemical stability window, directly influencing the energy and power densities of devices like supercapacitors, batteries, and conductive materials. Biopolymer-based gel electrolytes have emerged as a promising alternative to conventional petroleum-based materials due to their inherent properties, specifically, hydrophilicity, biocompatibility, mechanical robustness, thermal stability, and environmental friendliness. Recent studies have demonstrated the advantages of using biopolymers such as cellulose, chitosan, alginate, and gelatin, which not only enable efficient ion transport through their functional groups but also allow the integration of multifunctional properties like self-healing, stretchability, and temperature tolerance [155]. Among these, alginate stands out for its capacity to form complexes with a wide range of mono- to tetravalent cations, tuning the physicochemical behavior of the electrolyte and enabling its use across diverse fields. Furthermore, these biopolymers can mitigate key limitations in Zn batteries, such as hydrogen evolution, Zn corrosion, and dendrite formation. The fabrication of biopolymer-based electrolytes often employs environmentally friendly techniques. Examples include cross-linking, which can be established through either the external or internal gelation of the biopolymers, the use of composite matrices for the incorporation of micro- and/or nanostructures, and advanced manufacturing methods such as 2D or 3D printing technologies. These methods support scalability and reduce the ecological impact of gel electrolyte use. Performance evaluation metrics specific to the mechanisms of different types of supercapacitors have also been proposed, helping to standardize and advance the field [156,157]. However, significant efforts are still needed to enhance the key properties of biopolymer-based gel electrolytes, including their relatively low ionic conductivity at room temperature, electrochemical and thermal stability, and durability, and to address issues such as mechanical flexibility without compromising conductivity in order to aid the development of low-cost, scalable, and environmentally friendly methods for biopolymer extraction and electrolyte production [158]. In conclusion, the transition toward sustainable gel electrolytes is not only vital from an environmental perspective but also opens new opportunities for the design of high-performance, safe, and multifunctional flexible energy storage systems. Supported by recent publications, this measure underscores the need for continued innovation in bio-based materials and green fabrication strategies.

## 4. Conclusions

The integration of gel electrolytes into textile-based power sources presents a promising pathway for advancing flexible and wearable electronics. Compared to traditional liquid electrolytes, gel electrolytes offer significant advantages, including enhanced safety, leak resistance, mechanical flexibility, and improved interface compatibility. These attributes make them particularly well-suited for applications in sectors such as healthcare, sports, and fashion, where reliable and adaptable energy solutions are essential. Despite these advantages, challenges such as lower ionic conductivity and long-term stability must be addressed to fully optimize their performance. Regarding these restrictions, doped and hybrid gel electrolytes offer a promising approach to transforming conventional textiles into conductive and flexible materials for use in wearable electronics with improved properties. Recent advancements in gel electrolytes have related to enhancing ionic conductivity through the incorporation of various additives, namely inorganic nanoparticles, ionic liquids, carbon-based materials and polymer blends. Notably, MXene and PEDOT:PSS are two advanced materials that, when combined, have shown significant promise in enhancing the conductivity of gel electrolytes for textile applications. Despite these improvements, there are still a few challenges. In future research, we recommend the uniformization of units to ensure consistency and facilitate comparison across studies. The variability in textile substrates, fabrication methods, and testing conditions makes it difficult to directly compare performance metrics. As a result, there is an urgent need for unified test protocols and consistent reporting standards in the field. Continued research and innovation in material formulations, structural designs, and fabrication techniques will be crucial to overcoming these limitations. This review underscored the potential of gel electrolytes in various textile forms, including fibers, yarns, and woven or non-woven fabrics, and highlighted strategies for their effective integration. As advancements continue, the development of high-performance, durable, washable, and scalable gel electrolytes will be key to unlocking new possibilities in electronic textiles, paving the way for the design of next-generation wearable energy storage systems.

## Figures and Tables

**Table 2:** Comparison of yarn-based energy storage devices using gel electrolytes, highlighting preparation methods and electrochemical properties.

Gel Electrolyte	Yarn Composition	Electrodes	Preparation Method	Conductivity Tests and Results	Ref
PVA/KCl	PET	Au/Ni-MOF@ carbon yarn	PET dip-coating in dopamine; PET/PDA electroless plating; dip-coating in Ni-Ni_3_HHTP_2_ solution	Length capacitance of 1.1 × 10^2^ mF/cm; energy density of 3.9 × 10^−6^ Wh/cm; power density of 2.5 × 10^−4^ W/cm	[57]
PVA/ZnSO_4_	Stainless-steel CO blend	G/Zn-MnO_2_	MnO_2_ (cathode) or Zn (anode) electrodeposition PVA/ZnSO_4_ coating	Specific capacity of 43.53 mAh/g; energy density of 5.2 × 10^−2^ Wh/g	[58]
PVA/H_2_SO_4_	Cellulose	Ti_3_C_2_ MXene	Mxene dip-coating, followed by gel electrolyte coating	Conductivity up to 440.3 ± 0.9 S/cm; specific capacitance of 7.6 × 10^2^ mF/cm at 2 mV/s; 2000-cycle stability at ~14% compression strain	[59]
PVA/H_2_SO_4_	CO/Stainless steel	PEDOT:PSS	PEDOT in situ polymerization; SC assembled and woven	Areal specific capacitance reached a max. 1.6 × 10^2^ mF/cm^2^; Areal energy density 1.0 × 10^−5^ Wh/cm^2^; 81.9% capacitance retention after 10,000 cycles	[60]
PVA/H_2_SO_4_	Stainless steel/CO	PPy	PPY electrochemical deposition; gel electrolyte dip-coating	High areal capacitance (up to 3.4 × 10^2^ mF/cm^2^); high cycle stability (apx. 93% capacitance retention))	[61]
PVA/H_3_PO_4_	CO	PAN/PEDOT:PSS	Ni dip-coating; PAN electroless deposition; PEDOT:PSS dip-coating	Volumetric capacitance 2.7 × 10^4^ mF/cm^3^; energy density 9.6 × 10^−3^ Wh/cm^3^; power density 8.3 × 10^−1^ W/cm^3^	[62]
PVA/H_3_PO_4_	CO/Carbon fiber	Carbon fiber yarn	CO + carbon fiber twisting; yarns fixed into ABS mold; material coated with cellulose-based ionic hydrogel and gel electrolyte	Specific energy density 1.7 × 10^−8^ Wh/cm^2^; specific power density 5.3 × 10^−4^ W/cm^2^; 92% capacitance retention	[63]
PVA/H_3_PO_4_	MWCNT/ TiO_2_ fibers	MWCNT/ TiO_2_	TiO_2_ nanofibers (NFs) produced by electrospinning; TiO_2_ NF electrophoretic deposition in MWCNT; D gel electrolyte dip-coating; yarn twisting of 2 electrodes + gel electrolyte dip-coating	Good capacitance (3.7 × 10 mF/cm at 0.1 mA/cm); capacitance retention; good energy (1.2 × 10^−5^ Wh/cm) and power (3.7 × 10^−4^ W/cm) densities	[64]
PVA/H_3_PO_4_/KI	Carbon fibers	Carbon fibers	gel electrolyte dip-coating; yarn twisting of 2 electrodes; second dip-coating in gel electrolyte	Volumetric capacitance 363.9 F/L; energy density 5.1 × 10^−2^ Wh/L; specific capacitance (13.9 mF) using a 7.5 mM KI solution	[65]
PVA/H_3_PO_4_	CO and Nylon^®^	Ti_3_C_2_T_x_ MXene	MXene yarn coating of CO and Nylon^®^; yarn knitting in fabrics; PET yarn knitted in between; gel electrolyte coating	Capacitance up to 7.1 × 10^2^ mF/cm^2^ and 5.2 × 10^2^ mF/cm^2^, in 1 M H_3_PO_4_ and H_3_PO_4_ gel electrolyte; delivery energy 2.5 × 10^−5^; power densities 4.7 × 10^−7^ Wh/cm^2^; cycling stability	[66]
PVA/H_3_PO_4_	Carbon nanotubes (CNT)	PEDOT:PSS/CNT- *NMP*	CNT forest and sheet by chemical vapor deposition; NMP drop casting in CNT forest; yarn electrode production via biscrolling process; gel electrolyte coating	Capacitance 112.76 F/g; maximum power and energy density 9.8 × 10^2^ W/kg (1.2 Wh/kg) and 3.8 Wh/kg (1.5 × 10^2^ W/kg); cycling stable performance after 5000 cycles	[67]
PVA/KOH	PET	PPy@NiCo-double hydroxide@stainless-steel electrodes	NiCo-layer double hydroxide via hydrothermal process; PPU electrochemical deposition; gel electrolyte coating	Good specific capacitance 1196 F/g; energy density 6.6 mWh/cm^3^; power density 1.6 × 10^−1^ mW/cm^3^; good cycle performance (85.7% capacity retained)	[68]
PVA/KOH	Au/CO	NiCo2S4@Ni−Co LDH	Hydrothermal synthesis of NiCO_2_S_4_ nanotubes on Au/CO yarns; Ni−Co LDH nanosheets electrodeposition	Capacitance 5.7 × 10^3^ mF/cm; good areal energy density 3.5 × 10^−6^ Wh/cm^2^; stable cycling performance small capacitance retention decrease (9%)	[69]
PVA/KOH	PET	rGO/CNT and NiCo-BOH	Ni electroless deposition on PET yarns + Cu electrodeposition; co-self-assembled rGO/CNT hydrothermal reaction on Ni/Cu-plated PET yarns; gel electrolyte dip-coating	Energy density apx. 7.8 × 10^−5^ Wh/cm^2^; high power density 1.4 × 10^4^ W/cm^2^; stable cycling performance 82.7% capacity retention	[70]
PVA/KOH	Co wire + CO yarn	NiMnO3-rGO@CO-Cu	NiMnO_3_-rGO nanocomposites via hydrothermal reaction; CO yarns dip-coating in graphite and PVDF; Cu wire and CO yarns are woven; Co-Cu fibers dip-coating in NiMnO_3_-rGO; electrode dip-coating in gel electrolyte	Maximum specific capacitance 4.0 × 10^2^ mF/cm^2^; maximum specific capacitance 8.3 × 10 mF/cm^2^; specific energy density 17.5 µWh/cm^2^	[71]
PVA/KOH	Carbon-based yarns (CBY)	CoNi-layered double hydroxides@CBY and S-dopes carbon nanoparticles@CBY	CoNi-layered double hydroxide nanosheets; in situ growth via hydrothermal reactions; yarn anchoring assembly	Specific surface area 655 m^2^/g^1^ of CoNi-layered double hydroxides@CBY; voltage operating range 1.4 V; capacitance 2.3 × 10^2^ mF/cm^2^; energy density (6.2 × 10^−5^ Wh/cm^2^); stable cycle performance	[72]
PVA/KOH	CNT-coated CO (CCY)	NiCo-LDH@CCY	CO yars dip-coated in CNT dispersion; CCY dip-coating to produce NiCo-LDH@CCY; supercapacitor assembled by coating two yarns in a PET substrate with PVA/KOH or via PVA/KOH solution dip-coating	Good areal capacitance 1.24 × 10^2^ mF/cm^2^, current density 0.2 mA/cm^2^ and energy density 3.9 × 10^−5^ Wh/cm^2^	[73]
PVA/KOHNa_2_SO_4_	Transition metal oxide (TMO)-Ni-TMO trilayer nanoribbon yarn	(TMO)-Ni-TMO trilayer nanoribbon	TMO-Ni-TMO nanoimprinting in the mold; nanoribbon array delamination; twisting the nanoribbon array to form yarns; production of YSC, coated with gel electrolyte, using two TMO yarns or one TMO yarn + graphene fiber	Maximum energy density 7.6 × 10^−2^ Wh/cm^3^ achieved for graphene–CoNixOy@Ni (G-CNO) YSCs; power density 2.4 × 10^−1^ Wh/cm^3^ achieved with G-CNO YSCs; 94.2% initial capacitance retention after 10,000 cycles	[74]
PVA/H_2_SO_4,_ PVA/KOH, PAAm/NaCl	MWCNT	MWCNT	Floating catalyst chemical vapor deposition to obtain MWCNT, followed by twisting; insulators prepared by connecting two Ag wires to the MWCNT yarns; gel electrolyte added to the coils	High-recognition voltage signals (4–15 mV) with low noise (0.024 mV); high voltage amplitude sensitivity to tensile stretches in multiple frequencies (0.1–10 Hz) and strains (1–80%)	[75]
PVA/LiCl	CO	rGO	Polymer-assisted metal deposition (PAMD) of Ni on CO; rGO electrochemical deposition; yarn embroidering	Capacitance 1.6 × 10 mF/cm^2^ for in-plane MWNT/Ni-CO@fabric with PVA/LiCl at 0.8V; cycling performance—97% capacitance retention	[76]
ZnCl_2_/NH_4_Cl/CMC	Cellulose	PANI (cathode) Zn (anode)	Cellulose yarn electrospinning; PANI in situ polymerization; conductive slurry coating to obtain cathodes; Zn electrochemical deposition in conductive slurry-coated cellulose yarn to obtain anodes; cathode CMC coating, followed by twisting with anode; gel electrolyte coating by CMC swelling	Energy density 153.2 and 6.1 × 10 Wh/Kg; good cyclic stability, specific capacity maintained at 109.7 mAh/g	[77]

**Table 3:** Comparison of woven energy storage devices using gel electrolytes, highlighting preparation methods and electrochemical properties.

Gel Electrolyte	Fabric Composition	Electrodes	Preparation Method	Conductivity Tests and Results	Ref.
PVA/H_3_PO_4_- commercial fluorescent pigment of ZnS-Mn	CO	CNT	Dip–pad–dry process with CNT; redox-active additive in a solid-gel electrolyte	100% stability after 8000 cycles; energy density of 1.6 Wh/kg; potency density of 6.4 × 10^2^ W/kg; high specific capacitance of 4.37 F/g	[1]
PVA/LiCl	CO	PPy; TsOH	Carbonization of the fabric; in situ electrochemical deposition with PPy; immersion in electrolyte gel	Specific capacity of 5.0 × 10^2^ mF/cm^2^; 73.6% stability after 1000 cycles; energy density range of 1.18–0.68 mWh/cm^3^; high power density of 1.8–8.4 × 10^−3^ W/cm^3^; breaking strength of 5.27 N	[78]
Nanofibrillated cellulose/polyacrylamide; sodium polyacrylate	CO	Co; Cu; Ag; Ni	CO activated with PdCl_2_ (loading catalyst); electroless deposition with Co, Ni, Cu, and Ag	Conductivity of ~200 kS/m; capacity of 1.0 mAh/cm^2^; high energy density of 1.7 m × 10^−3^ Wh/cm^2^; power density of 8.5 × 10^−3^ W/cm^2^; discharge capacity of 132 mAh	[79]
PVA/H_2_SO_4_	CO	PPy; PU; water repellent; 5-sulfosalicylate; ferric chloride	Spray-coating of CO fabric with PU and thickener; oxide polymerization with PPy; gel electrolyte coating	Stability of 85% after 3000 cycles; energy density of 9.0 × 10^−8^ Wh/cm^2^; power density of 1.9 × 10^−5^ W/cm^2^	[80]
PVA/KOH	CO	Graphene; Co(NO_3_)_2_.6H_2_O; Ni(NO_3_)_2_.6H_2_O; PANI; cetyl trimethyl ammonium bromide; PANI nanotubes	Chemical deposition with graphene/PANI nanotubes solution; immersion into NiCo/cetyl trimethyl ammonium bromide solution; gel electrolyte coating	Specific capacitance of 4.3 × 10^2^ mF/cm^2^; 84.05% stability after 10,000 cycles; 15.95% deterioration after 10,000 cycles; specific surface area of 30.63 m^2^/g and pore volume of 0.176 cm^3^/g; surface roughness of 98.7 nm; energy density of 8.0 × 10^−5^ Wh/cm^3^	[81]
PVA/H_2_SO_4_	CO	PEDOT; Ti_3_C_2_T_x_ MXene	Vapor phase polymerization with PEDOT; spray-coating with MXene dispersion; immersion into gel electrolyte	Sheet resistance of 3.6 Ω/sq; specific capacitance of 1.0 × 10^3^ mF/cm^2^; areal energy density of 1.3 × 10^−5^ Wh/cm^2^; strong joule heating performance of 193.1 ºC; 36.62 dB EMI shielding effectiveness; high sensitivity; 25.7% capacitance retention	[82]
TEABF_4_-polyacrylamide	CO	Carbon black; activated carbon; PVA; 1,2,4-Trichlorobenzene	Spraying with ink formulation (carbon black, activated carbon, poly(ethylene-co-vinyl alcohol), and 1,2,4-Trichlorobenzene); impregnation with gel electrolyte	Specific capacitance of 3.4 × 10 mF/cm^2^; energy and power densities of 18.9 μW h/cm^2^ and 2.4 × 10^−4^ W/cm^2^; specific surface area of 1874.2 m^2^/g and pore size of 1.6–2.7 nm; 48% capacitance retention after 2 mouths of aging; 88% Coloumb efficiency for 2 mouths of aging assay	[83]
PVA/H_2_SO_4_	CO	PPy; PVA-co-ethylene nanofiber suspension	Spray-coated on the fabric with PVA-co-etthylene nanofiber suspension; in situ chemical polymerization with PPy layers; gel electrolyte coating	100% capacitance retention after 10,000 cycles; 98.17% stability over 1000 bending cycles; specific capacitance of 6.7 × 10^2^ mF/cm^2^; areal energy density of 6.0 × 10^−5^ Wh/cm^2^; 90–100% Coloumb efficiency	[84]
PVA/KOH	CO	NiCl_2_·6H_2_O; CoCl_2_·6H_2_O; Fe_3_C	Electroless plating process (CO fabric/Ni:Co); sandwiched with gel electrolyte and silk fabric as separator	High specific capacitance of 113.7 C/g; 80% stability after 4000 cycles; energy and power densities of 4.7 × 10 Wh/kg and 1.5 × 10^3^ W/kg	[85]
PVA/KOH	CO	ZnO nanoparticles; CuS microsphere	Dyeing of CO fabric; atomic layer deposition of ZnO nanoparticles; carbonization process; hydrothermal reaction with CuS; gel electrolyte immersion	Specific capacitance of 1.8 × 10^3^ mF/cm^2^; 85.2% stability after 5000 cycles; energy and power densities of 0.27 Wh/cm^2^ and 4.3 × 10 W/cm^2^	[86]
PVA/KOH	CO	Procion reactive dye; cellulose and polyethylene terephthalate films	Dyeing and carbonization of CO fabrics; dipping in the electrolyte; supercapacitor sandwiched with cellulose and polyethylene terephthalate films	Specific surface areas of 448.2–622.1 m^2^/g and pore volumes of 0.22–0.39 cm^3^/g; specific capacitance of 1.1 × 10^3^–1.8 × 10^3^ mF/cm^2^; 79.3% capacitance retention; sheet resistance of ∼30–45 Ω/sq; 92.2% stability after 5000 cycles; volumetric specific capacitance of 1.8 × 10^4^ mF/cm^3^; energy density of 5.7 × 10^−3^ Wh/cm^3^ at the power density of 4.5 × 10^−3^ W/cm^3^	[87]
PVA/KOH	CO	NiCl_2_·6H_2_O; Na_2_WO_4_·2H_2_O	Thermal annealing with NiCl_2_·6H_2_O and Na_2_WO_4_·2H_2_O; sandwiched with gel electrolyte	High specific capacitance of 60.61 F/g; 95% stability after 3000 cycles; 99.7% stability after bending; energy and power densities of 2.3 × 10 Wh/kg and 8.3 × 10^2^ W/kg	[88]
LoSalt^®^/ Grenade Energy^®^/Shopper isotonic drink + agar and k/carrageenan	CO	Carbon black; activated carbon; ethylene-vinyl acetate; 1,2,4-Trichlorobenzene	Spray-coating with ink formulation; gel electrolyte impregnation	Specific capacitance of 2.3 × 10 mF/cm^2^; energy and power densities of 2.3 × 10^−6^ W h/cm^2^ and 2.0 × 10^−4^ W/cm^2^	[89]
PVA/LiCl	Cellulosic	Dopamine; GO	Dopamine in situ polymerization; immersion on GO solution; carbonization; gel electrolyte immersion	Specific capacitance of 1.2 × 10^3^ mF/cm^2^; specific area of 347.6 m^2^/g; 6% stability after 4000 bending cycles	[90]
PVA/H_2_SO_4_	CO	PPy; GO; Ag	Vacuum filtration of GO; addition of PPy and Ag^+^ through UV-induced in situ polymerization; gel electrolyte immersion	Specific capacitance of 1.7 × 10^3^ mF/cm^2^; capacitance retention of 46.9%; energy potency of 1.1 × 10^−3^ Wh/cm^2^; 90.5% stability after 10,000 cycles; 89.7% stability at 180º after 10,000 bending cycles	[91]
PVA/Zn(OTf)_2_	CO	VO_2_; Zn nanosheets; CNT film; H_2_C_4_O_4_·2H_2_O; H_2_O_2_; ethanol; PDMS	Carbonization of fabrics; O_2_ plasma treatment on fabric; VO_2_ mixture with carbonized CO fabric; pressure sensors production; gel electrolyte immersion; sandwich structure containing Zn/CNT film (anode) and VO_2_/carbonized CO (cathode)	Flexible pressure sensor: electrical conductivity of ∼95.8 Ω/sq; tensile stress of ∼6.7 MPa; tensile strain of 155%; sensibility of 0.07–7.12 kPa^−1^; response/recovery time of 12 and 8 ms Aqueous Zn-ion batteries: specific capacity 301.5 mAh/g; 99.8% Coulombic efficiency; 88.7% stability after 5000 cycles	[92]
PVA/KOH	CO	MWCNT; thermoplastic PU	Gel electrolyte applied through dip–pad–dry method; screen-printing with MWCNT ink; multiple layers of electrodes and fabric separators were sandwiched	High resistance of ∼120 Ω; specific capacitance of 4.17 mF/cm^2^; high bending strain of 20%/s; ∼97.4% stability after 1000 cycles	[93]
PVA/KOH	CO	Ag; CNT; graphene	Screen-printing with coating ink (Ag, CNT/graphene, and textile ink); gel electrolyte immersion	Specific capacitance of 6.8 × 10^2^ mF/cm^2^; 80% stability after 3000 cycles	[94]
PVA/KOH	CO	Ag; activated carbon; graphene; CNT	Screen-printing with coating ink (Ag and activated carbon); gel electrolyte immersion	Specific capacitance of 3.3 × 10^3^ mF/cm^2^; ∼130% stability after 10,000 cycles; high energy and power densities of 5.1× 10^−4^ Wh/cm^2^ and 1.5 × 10^−1^ W/cm^2^	[95]
PVA/KOH	CO	MWCNT; thermoplastic PU; NMP	Textile electrodes printed using MWCNT ink; gel electrolyte coating	Specific capacitance of 1.4 × 10 mF/cm^2^; high bending strain of 20%/s; 90% stability after 2000 cycles	[96]
PVA/H_2_SO_4_	CO	Graphene; carboxymethyl celulose; PU	Printing of graphene ink onto textiles; gel electrolyte drop-casting	Specific capacitance of ∼3.2 mF/cm^2^; resistance of 30 Ω/cm; energy and power densities of 2.8 × 10^−4^ Wh/cm^2^ and 3.0 × 10^−3^ W/cm^2^; stability ∼10,000 cycles; durability of 3.5-time higher resistance after 10 washing cycles; device retention of 95% after 10,00 cycles	[97]
PVA/LiCl	CO	N-doped carbon; FeO_4_; PPy	Impregnation with PPy; oxidative polimerization with FeCl_3_; carbonization; gel electrolyte immersion	Specific capacitance of 135 F/g; moderate specific surface areas (700.8 m^2^/g); rate capability of 1–10 mV/s (44.4%); 88.4% stability after 1000 cycles; 92.3% Coulombic efficiency after 1000 cycles	[98]
PVA/H_3_PO_4_	CO	PEDOT:PSS; dimethyl sulfoxide; MXene, graphene nanoscroll; PPy	PEDOT:PSS dip-dry coating; dimethyl sulfoxide immersion; one-step in situ polymerization with MXene, graphene nanoscroll, and PPy; gel electrolyte immersion	Capacitance of 2.7 × 10^3^ mF/cm^2^; high energy density of 3.2 × 10^−4^ Wh/cm^2^ and power density of 4.6 × 10^−4^ W/cm^2^; 79% stability after 2500 cycles; 97.8% stability after bending; 92% waterproof property after 2 h	[99]
PVA/H_3_PO_4_	CO	MWCNT; sodium dodecylbenzenesulfonate	Dip–pad–dry with oxidized MWCNT dispersion; gel electrolyte immersion	High energy density of 3.5 Wh/kg; 98% stability after 5000 cycles; specific capacitance of 3.91 F/g	[100]
PVA/H_3_PO_4_	CO	MWCNT; sodium dodecylbenzenesulfonate	Dip–pad–dry with MWCNT; gel electrolyte coating	Specific capacitance of 9.2 × 10^3^ mF/cm^2^; 96.3% stability after 5000 cycles; energy and power densities of 6.3 Wh/kg and 1.1 × 10^3^ W/kg	[101]
PVA/LiCl	CO	MXene	Exfoliating; delaminating; impregnation with MXene suspensions; carbonization; gel electrolyte immersion	Specific capacitance of 5.0 × 10^2^ mF/cm^2^; 74% stability after 10,000 cycles; hydrophilic properties; electric conductivity of 5882 S/m	[102]
PVA/KCl	CO	Cu-MOFs; HAuCl_4_; polydopamine; polyethylene terephthalate film	Dopamine hydrochloride immersion; immersion in HAuCl_4_; immersion in Cu-MOF solution; gel electrolyte immersion	Specific capacitance of 258 F/g; energy density of 4.3 × 10^−4^ Wh/cm^2^; 83% stability after 3000 cycles; 94% Coulombic efficiency after 3000 cycles	[103]
PVA/H_3_PO_4_	CO	PPy; PANI; PEDOT; CuCl_2_	CuCl_2_ padding; vapor phase polymerization in situ with monomer (PANI, PEDOT or PPy) in ice bath; gel electrolyte immersion	Specific capacitance of 9.0 × 10^2^ mF/cm^2^; 86.5% stability after 12,000 cycles; 90% stability at 180º after 1000 bending cycles	[104]
PVA/KOH	CO	CuS nanosheets	Carbonization and oxidation of the CO fabric; electrodeposition with CuS nanosheets; gel electrolyte immersion	Specific capacity of 1.3 × 10^3^ mF/cm^2^; 91.8% stability after 2000 cycles; ultrahigh energy density (0.96 Wh/cm^2^); power density of 4. 4 × 10^3^ W/cm^2^	[105]
PVA/H_2_SO_4_	CO	Au nanoparticles; tetraoctylammonium bromide; PANI; PDMS	Layer-by-layer deposition of tetraoctylammonium bromide; PDMS dip-coating eletrodeposition of PANI; gel electrolyte deposition	Au/PDMS: hydrophobicity (120–140°); washing fastness after 60 cycles; resistance to high-frequency ultrasound (300 s); high breathability; corrosion resistance; self-cleaning capability for 10 cycles; 98.88% cleaning efficiency Au/PANI: areal capacitance 5.0 × 10^2^ mF/cm^2^; energy 3.3 × 10^−5^ Wh/cm^2^ and power 1.1 × 10^−5^ Wh/cm^2^ density; 77% stabilty after 1000 cycles	[106]
PVA/KOH	CO	Cu; Ni	Sputtering (Cu); electrochemical deposition (Ni); electroplated; gel electrolyte immersion	Specific capacity of 243.9 μAh/cm^2^; 70% stability after 5000 cycles; energy and power densities of 4.9 × 10 Wh/kg and 3.9 × 10^2^ W/kg	[107]
PVA/KOH	CO	Nickel tungstate; niquel oxide; Ni(NO_3_)_2_	Ultrasonic spray-coating method with Ni(NO_3_)_2_; in situ chemical synthesis of a uniform nickel oxide layer; electrochemical deposition of nickel tungstate; gel electrolyte immersion	Specific capacity of 1.4 × 10^2^ mF/cm^2^; high specific of energy 12 μWh/cm^2^; specific power of 6.9 × 10^−8^ W/cm^2^; 78% stability after 5500 cycles	[108]
PVA/KOH	CO	Zn(NO_3_)_2_+MOFs+CoNi + layered double hydroxides (cathode); Zn-N (anode)	Zn(NO_3_)_2_ immersion; carbonization; Co-MOFs, NiSO_4_⋅6H_2_O and layered double hydroxide deposition; gel electrolyte application	Discharge capacity retention (60.11% after 1000 cycles); 98% Coulombic efficiency; bending resistance; high specific capacitance (161.25 F/g); energy (4.7 × 10 Wh/kg) and power (2.7 × 10 Wh/kg) density	[109]
PVA/KOH	CO	Co-zeolitic imidazole framework-67 nanoparticles	Co-zeolitic imidazole framework-67 nanoparticles in situ deposition; carbonization; gel electrolyte application	Specific capacitance of 288.62 F/g; energy density of 1.6 × 10 Wh/kg; power density of 6.5 × 10 W/kg; bending resistance; 76.4% stability after 2000 cycles	[110]
PVA/H_2_SO_4_	CO 95%; spandex 5%	PANI;carbon;textile	Tandem procedure by imersing the fabric in acidic aniline solution; reaction with ammonium persulfate and drying the fabric at 60 °C, using H_2_SO_4_ as dopand and carbonizing assistant	Specific capacitance of 3.9 × 10^2^ mF/cm^2^; over 70% capacitance retention after 5000 cycles; energy density of 3.6 × 10^−2^ Wh/m^2^ at 7.5 × 10^−1^ W/m^2^ power density; stable capacitance under bending (0–180°) and stretching (up to 50% elongation)	[111]
PVA/H_2_SO_4_	CO 95%; spandex 5%	PANI;graphen;textile;HCl	Dipping and drying method, followed by in situ polymerization of aniline	Specific capacitance of 1.6 × 10^3^ mF/cm^2^; over 75% capacitance after 10,000 cycles; energy density of 7.6 × 10^−1^ Wh/m^2^; stable capacitive performance under bending from 0 to 180°; 77% retention over 600 bending cycles	[112]
PVA/KCl	Polyamide	Metal-coated textiles	Printing a graphite with ethyl cellulose as binder and terpineol as solvent; gel electrolyte sandwiched between the positive and negative electrode with piece of cellulose PET cloth as the separator	Areal capacitance of 3.2 × 10 mF/cm^2^ and an energy density of 2.8 × 10^−6^ Wh/cm^2^	[113]
PVA/KCl	Metal-coated polyamide;PET	Metal-coated textiles	Commercial metal-coated fabrics compared with a metal-free graphite coated PET/cellulose fabric	Ni/Cu-coated PET fabric: capacitance 9.9 × 10 mF/cm^2^; energy density of 8.8 × 10^−6^ Wh/cm^2^ Ni/Cu/Ag-coated polyamide: capacitance 4.7 × 10 mF/cm^2^ at 5 mV/s; energy density of 4.2 × 10^−6^ Wh/cm^2^; stable performance over 5000 charge–discharge cycles	[114]
KOH	PET; CO blend	NiCoAl-LDH); Ti3C2Tx; MXene; Ag nanowires as positive electrode; active carbon (negative electrode)	Interdigital pattern obtained by printing and electroless deposition; anchoring of the battery-type material onto conductive MXene and hydrothermal treatment; active carbon inks deposited by screen-printing; flexible supercapacitor assembled by covering a layer of PVA/KOH gel electrolyte, and encapsulated by scotch tape	Positive electrode: capacity 592 C/g, excellent rate performance and cycling stability over 10,000 cycles Positive electrode and negative electrode: energy density of 2.2 × 10^−5^ Wh/cm^2^ and a power density of 3.0 × 10^−3^ W/cm^2^	[115]
PEO/KOH	PET	Silver oxide	Stencils of different thicknesses used to print different layers of the battery in polyamide-nylon 6 and PET	Capacity of 0.6 mAh/cm^2^ with an active electrode area of 0.5 cm × 1 cm	[116]
PVA/H_2_SO_4_	PET	rGO nanosheets; PPY	Dipping and drying method	Capacitance of 2.3 × 10^3^ mF/cm^2^; volumetric capacitance of 5.5 × 10^3^ mF/cm^3^; energy density of 1.1 × 10^−5^ Wh/cm^2^; power density of 3.0 × 10^−5^ W/cm^2^; retains 76% of its initial capacitance after 6000 cycles and mechanical stability under bending	[117]
PVA/Na_2_SO_4_	Cu; Ni coated conductive PET	PPy–graphene–PPy-coated fabric	Sandwich configuration with PVA/Na_2_SO_4_ as a gel electrolyte and filter paper as a separator; electrodes and filter paper immersed in the gel electrolyte before being assembled into a supercapacitor device	Capacitance of 6.8 × 10^2^ mF/cm^2^; energy density of 6.4 × 10^−5^ Wh/cm^3^; power density of 0.6 × 10^−3^ W /cm^3^; maintained 94.2% of its capacitance after 4000 cycles	[118]
PVA/H_2_SO_4_	CO; PET	Graphene; microcircuit encapsulant PE773	Pad–dry–cure method with graphene ink and encapsulation (microcircuit encapsulant PE773); immersion in gel electrolyte	Specific capacitance of ∼2.7 mF/cm^2^; capacitance retention of 98% after 150 cycles at 180º flexion; high domestic washing fastness (10 cycles)	[119]
PVA/H_3_PO_4_	Silk	CSF; PPy	Carbonization of silk fabrics; potentiostatic electrodeposition of PPy; immersion of the electrodes in gel electrolyte; SC assembly using cellulose sandwich	Composite: capacitance of 4.0 × 10^3^ mF/cm^2^ and cycling stability of 88.6%; capacitance retention after 1500 cycles. SC: areal specific capacitance of 6.7 × 10^2^ mF/cm^2^; energy density of 6.9 × 10^−3^ Wh/cm^3^	[120]
PVA/H_3_PO_4_	Nylon^®^	CNT; Nylon^®^	Nylon^®^/CNT electrodes produced by dip-coating; Nylon^®^/rubber:Nylon^®^/CNT laminates production; SC production using a Nylon^®^ sheet as separator	Capacitance of 117 F/g (at 2 mV/s); maximum energy density of 4.0 Wh/kg	[121]
PVA/H_3_PO_4_	Silk	PANI@GO	Dip-coating of silk in the respective mixture (either GO or PANI). For GO@PANI-coated fibers, the GO-coated silk materials were dipped in the PANI mixture.	Specific capacitance of 450 F/g; capacitance of 71.2 F/g obtained with the symmetrical PANI@GO-SL/PVA/PANI@GO–silk capacitor; 87.4% capacity retention at 5000 cycles; energy and power densities of 2.5 × 10 Wh/kg and 8.0 × 10^3^ W/kg	[122]
PVA/H_3_PO_4_	PP; PET; PAN	rGO	Reactive inkjet printing of GO in the fabric with concomitant reduction; dip-casting of the gel electrolyte; all-solid-state SC assembly	PP fabric: specific capacitance of 1.3 × 10 mF/cm^2^; power and energy densities of 4.6 × 10^−3^ W/cm^2^ and 1.2 × 10^−3^ Wh/cm^2^; apx. 100% of its original capacitance after 5000 cycles	[123]
PVA/H_3_PO_4_	Polyamide; carbon fibers	PEDOT:PSS	Weaving a polyamide warp yarn around the self-designed mode; interwoven weft yarn (polyamide yarn/Ag-coated polyamide yarn); supercapacitor assembled by using PEDOT:PSS-coated carbon fibers, coated with gel electrolyte and separated by cellulose	Capacitance 1.3 × 10 mF/cm^2^ (79.9 F/g) at a current density of 0.1 mA/cm^2^; power and energy densities of 4.6 × 10^−3^ W/cm^2^ and 1.2 × 10^−3^ Wh/cm^2^	[124]
PVA/H_2_SO_4_	Kevlar^®^	rGO	rGO-coated Kevlar^®^ fibers produced by modified hydrothermal gelation; dip-coating of rGO@Kevlar cloth in gel electrolyte	rGO@Kevlar^®^ fibers (38.1% rGO): specific strength of 1.6 MPa.m^3^/kg; specific capacitance of 57 F/g rGO@Kevlar^®^ cloth SC. Withstands impact of 9.1 N and deformation of 90º.	[125]
PVA/KOH-Zn(Ac)_2_- LiOH- Ca(OH)_2_	PET; polyamide 6,6	Zn; Cu; NiCo	Kapton applied on fabric Ni and Cu deposited by electroless and electrodeposiition; 2 interdigitated Cu electrodes coated; Zn and NiCo BOH nanosheets were electrodeposited; coating with gel electrolyte	Electroplated Zn anode and a Ni cathode; energy density of 2.6 × 10^2^ Wh/kg; power density of 1.0 × 10^4^ W/kg; stable cycling performance of 82.7% for 1500 cycles; good mechanical reliability (bending, twisting and tailoring)	[126]
PVA/LiCl	Bamboo	MnO_2_–NiCo_2_O_4_; rGO	Printing of Ni-Co + printing of KMnO_4_ for anode; printing with rGO + hydrazine reduction for cathode; device prepared using an anode and cathode sheets, separated by a bamboo fabric sheet, and coated with gel electrolyte	MnO_2_–NiCo_2_O_4_/rGO device shows stable performance within a 0–1.6 V range; capacitance of 2.1 × 10^3^ mF/cm^2^; energy density of 3.8 × 10^−2^ W/cm^3^; power density of 2.7 W/cm^3^; 92% of capacitance retention after 5000 cycles and low charge transfer resistance 3.2 Ω	[127]
PVA/KCl	Ag-coated polyamide (Berlin fabric)	Ag-coated polyamide; graphite	Printing coated electrodes and gel electrolyte	Areal capacitance 1.3 × 10 mF/cm^2^	[128]
PVDF/LiTFSI	PP-based satin	LFP/LTO;carbon black	Thermally drawn fibers comprising anode (LTO), cathode (LFP), gel electrolyte (PVDF/LiTFSI) and conductive polymer (carbon black). The fibers produced are then woven into the satin.	Battery discharge capacity of apx. 123 mAh and discharge energy of apx. 2.2 × 10^−1^ Wh. Woven: 96% of capacity retention after 1000 bending cycles.	[129]

**Table 4:** Comparison of CC-based energy storage devices using gel electrolytes, highlighting preparation methods and electrochemical properties.

Gel Electrolyte	Textile Composition	Electrodes	Preparation Method	Conductivity Tests and Results	Ref.
PVA/NH_4_Cl-ZnCl	3D bicontinuous porous carbon-sheathed carbon cloth (CC–PC)	CCPC@PANAC (cathode); CC@Zn NP (anode)	PANAC cathode produced by the copolymerization of PANI with a redox-active phenothiazine derivative. The anode constructed by depositing Zn nanoplate arrays onto CC by the electrochemical method. The gel electrolyte is placed between the electrodes.	Energy density of 3.5 × 10^2^ Wh/kg; specific capacity of 306.3 mAh/g; capacitance retention of 86.6% after 2000 bending cycles	[130]
LiCl/PVA	CC	NHPCN@CC (electrodes)	NHPCN@CC was obtained by self-assembly of a sol–gel MSS template onto CC, followed by PDA coating and subsequent carbonization. The electrodes were immersed in a LiCl/PVA gel electrolyte.	Energy density of 1.0 × 10 Wh/kg (at 1.0 × 10^4^ W/kg) and 2.4 × 10 Wh/kg (at 5.0 × 10^2^ W/kg); capacitance retention of 85% (for 8000 cycle)	[131]
PVA/KCl	CC	Flexible conductive porous electrode; PANI/ZnO@ZIF-8-CC	PANI/ZnO@ZIF-8-CC electrode: in situ growth of hollow ZnO spheres on activated CC; core–shell structure, created by coating the ZnO core with a ZIF-8 shell; aniline electropolymerization used to deposit a homogeneous PANI coating on both the inner and outer surfaces of ZnO@ZIF-8-CC	Areal capacitance of 4.8 × 10^3^–4.0 × 10^3^ mF/cm^2^ (at 5–30 mA/cm^2^); energy density of 1.4 × 10^−4^–8.9 × 10^−5^ Wh/cm^3^; equivalent series resistance of 1.22 Ω; specific capacitance of 4.3 × 10^3^ mF/cm^2^; capacitance retention of 7% for 10,000 cycles	[132]
PVA/H_2_SO_4_-Fe^3+^-Fe^2+^	CC	PANI; CNTs core–shell (hybrid electrode)	PANI/CNTs@CC electrodes were obtained by the deposition of CNTs onto CC via LPCVD, followed by PANI coating through electropolymerization. The gel electrolyte was prepared using H_2_SO_4_, PVA, FeSO_4_·7H_2_O, and Fe_2_(SO_4_)_3_. The SCs were assembled by pressing.	Diffusion resistance of 0.236 Ω (PANI/CNTs); specific capacitance of 6.0 × 10^2^ mF/cm^2^ (at 5 mV/s); energy density of 2.3 × 10 Wh/kg (at a power density of 7.0 × 10^2^ W/kg); capacitance retention of 97% (after 2000 cycles)	[133]
NaClO_4_ /PVA	CC	MnO_2_ nanowires/CC and activated carbon	MnO_2_ nanowires/CC electrode prepared on CC by dip-coating and autoclave; the supercapacitor is assembled using both MnO_2_ nanowires/CC and activated carbon fibers, coated with the gel electrolyte.	Capacitance retention of 81% after 25,000 cycles while exposed directly to axternal enrivronment; consistent and stable charge storage between −40 and 40 ºC.	[134]
PVA/Na_2_SO_4_; PVA: EMIBF_4_	CC	Sm-Mo-C5 woven carbon fibers (WCFs)	SmVO_4_ nanoparticles were synthesized via hydrothermal techniques, using Sm(NO_3_)_3_·6H_2_O and NH_4_VO_3_. The SmVO_4_-MoS_2_ and CNT-SmVO_4_-MoS_2_ nanocomposites were synthesized by the same method. The supercapacitor was assembled by Vacuum-Assisted Resin Transfer Molding.	Specific capacitance of 1.0 × 10^3^ mF/cm^2^ (current density of 2.187 mA/cm^2^) in a three-electrode system. The SCs show specific capacitance of 2.9 × 10^2^ mF/cm^2^ (at a current density of 2 A/cm^2^). Capacitance retention: 72.5% to 50.000 cycles; maximum energy density of 8.0 × 10 Wh/Kg (at a power density of 1.0 × 10^3^ W/Kg).	[135]
FeDPCL (self-healing hydrogel electrolyte)	CC	PANI-CC	PANI@CWF electrodes were obtained by in situ electropolymerization of aniline onto carbon fabric. Hydrogel was prepared using AA, CTAB, C18, Fe(NO_3_)_3_·6H_2_O, and potassium persulfate. The gels were immersed in H_2_SO_4_. The eletrodes were directly paved on hydrogel eletrolyte without separator.	Energy density of 1.9 × 10 Wh/kg (at power densities of 6.7 × 10^2^ W/kg); capacitance retention of 86% (after healing behavior).	[136]

**Table 5:** Comparison of knitted fabric-based energy storage devices using gel electrolytes, highlighting preparation methods and electrochemical properties.

Gel Electrolyte	Structure and Composition	Electrodes	Preparation Method	Conductivity Tests and Results	Ref.
PVA/H_2_SO4	CO;CNT	CNT; PPy	Dip-coating pyrrole; supercapacitor assembled with two fabrics as electrodes, a CO fabric as a separator, and PVA/H_2_SO_4_ as the gel electrolyte	Areal capacitance of electrode of 4.1 × 10^3^ mF/cm^2^; quasi-rectangular curve cyclic voltametry; 93% Coulombic efficiency; 89% under bending	[137]
PAM/KOH	Spandex knitted fabric	Ni@NiCoP; SWCNT ST	Ni@NiCoP@SWCNT ST dip-coated in Ni@NiCoP ST into SWCNT ink; PAM-based hydrogel immersed in KOH; a layer of Ni@NiCoP@SWCNT ST and a layer of Ni@NiCoP ST attached to asymmetric electrodes in the supercapacitor; in between PVA/H_2_SO_4_ gel electrolyte	Eletrode conductivity 532 S/cm; areal capacitance 8.8 × 10^2^ mF/cm^2^; gravimetric capacitance 713 F/g; 101% retention after 6000 cycles	[138]
PVA/H_2_SO_4_	Cellulose	GNS; PEDOT:PSS	Sprayed GNS and PEDOT; gel electolyte coating	Specific areal capacitance of 2.4 × 10^2^ mF/cm^2^; energy density of 2.2 × 10^−5^ Wh/cm^2^; 83.9% capacitance after 10,000 cycles	[139]
PVA/H_3_PO_4_	Diamond-shaped warp knitting grid structure (stainless-steel mesh-SSM)	PEDOT; RGO; SSM	Two-step electrodeposition of PEDOT/RGO@SSM process in; PVA/H_3_PO_4_ gel electrolyte;	Areal capacitance of 5.3 × 10 mF/cm^2^; ~73% capacitance after 5000 cycles; rate capability of 3.6 × 10 mF/cm^2^; ~78% capacitance retention at 10% strain for 500 stretching cycles	[140]
PVA/LiCl	PET 90%; spandex 10%	Ni/rGO	Ni-coated textile was immersed in a GO dispersion for a hydrothermal reaction; PVA/LiCl gel electrolyte coating	Areal capacitance of 5.1 × 10 mF/cm^2^; no degradation at 50% of tensile strain; capacitance retention of 85.3% retention after charging/discharging for 5000 cycles; slightly slopped rectangular shape ciclic voltametry	[141]
ACN/PC/PMMA/LiClO_4_	Knitted Fabric (Nylon^®^ 82%; spandex 18%)	MWCNT; MoO_3_	MWCNT/MoO_3_ nanocomposite spray-coated over knitted textile; gel electrolyte	100% Coulombic efficiency; 86 and 76% capacitance after 5000 and 10,000 charge/discharge cycles; quasi-rectangular-shaped cyclic voltametry; areal capacitance of 3.4 × 10 mF/cm^2^	[142]
PVA/ H_2_SO_4_	CO	PPy	Dip-coating for in situ polymerization of PPy with, iron nitrate, and 5-sulfosalicylic acid e; gel electrolyte coating; device assembled simetrically	Areal capacitance of 4.5 × 10^2^ mF/cm^2^; energy density of 0.4 Wh; 30% capacitance after 500 cycles; ~84–160% retention rate during 1000 stretching procedures	[143]
KOH/Na_2_SO_4_	Knitted graphite fabric	Graphite and Mn-Cu alloys	Graphite fibers coated with Mn–Cu alloys by electrodeposition in an electrolyte solution	Specific capacitance of 9.3 × 10^4^ mF/cm^2^; areal capacitance of 9000 mF/cm^2^	[144]

**Table 6:** Comparison of non-weaving-based energy storage devices using gel electrolytes, highlighting preparation methods and electrochemical properties.

Gel Electrolyte	Non-Woven Composition	Electrodes	Preparation Method	Conductivity Tests and Results	Ref.
PVA/H_2_SO_4_	Graphene	Porous graphene fiber-assembled fabric	Micro wet spinning	Capacitance of 1.2 × 10^3^ mF/cm^2^; 100% for 60,000 cycles; energy density of 1.2 × 10^−4^ Wh/cm^2^	[147]
PVA/H_2_SO_4_	MXene-based fiber fabrics	MXene (Ti_3_C_2_Tx) graphene quantum dots	Micro wet spinning	Capacitance of 1.8 × 10^6^ mF/cm^3^; 100% stable for 5000 cycles; energy density of 2.1 × 10^−2^ Wh/cm^3^	[148]
PVA/H_2_SO_4_	rGO enveloped with PANI	rGO enveloped with PANI	Wet spinning	Capacitance of 3.8 × 10^3^ mF/cm^2^; 100% for 10,000 cycles; energy density of 4.9 × 10^−3^ W/cm^2^	[149]
Lithium perchlorate-PCL	PCL nanofibers containg silicon dioxide nanoparticles	PEDOT:PSS screen-printing on indium tin oxide coated polyethylene terephthalate	Electrospinning	Ionic conductivity of 5.2 × 10^−3^ S/cm; 100% for 100 cycles	[150]
Polyvinylidene fluoride/MgClO4/propylene carbonate	PVDF nanofibers	PVDF nanofibers	Electrospinning	Capacitance of 3832 mAh/cm^3^; ionic conductivity of 1 × 10^−3^ S/cm	[151]
Poly (ethylene oxide)	Poly (ethylene oxide)	Li-ions	Solvent casting and dipping	Run stably > 750 h at 0.5 mA/cm^2^; good security; 300 cycles with 1 mA/cm^2^	[152]
PVA/H_2_SO_4_/KOH	Pure cellulose	Carbon paper	Dipping	Capacitance of 1.4 × 10^2^ mF/cm^2^; 100% stable for 1000 cycles at 180° bending	[153]
PEGDA; ETPTA; liquid electrolyte; AIBN	PEGDA; ETPTA; liquid electrolyte; AIBN	Li (anode); LiPF_6_ (cathode)	Injection	Ionic conductivity 0.87 mS/cm; 100% for 200 cycles; resistance of 275 Ω	[154]

## Data Availability

No new data were created or analyzed in this study.
